# Propionate serves as a degradable control agent of citrus canker by acidifying cytoplasm and depleting intracellular ATP in *Xanthomonas citri*

**DOI:** 10.1128/mbio.00642-25

**Published:** 2025-04-29

**Authors:** Chaoying Liu, Jingtian Zhang, Meirui Song, Xiaoli Wang, Weiwei Lv, Xiaojun Ding, Junan Zhu, Yunfei Deng, Yifei Ge, Jian Wu, Utpal Handique, Shuo Duan, Yue Shen, Feng Luo, Shi Xiao, Xiaofeng Zhou

**Affiliations:** 1 State Key Laboratory of Biocontrol, Guangdong Provincial Key Laboratory of Plant Resources, School of Agriculture and Biotechnology, Sun Yat-sen University 582261 , Shenzhen, Guangdong, China; 2 Potato Engineering & Technology Research Center, Inner Mongolia University 12576 , Hohhot, Inner Mongolia, China; 3 National Navel Orange Engineering Research Center, Gannan Normal University 12450, Ganzhou, Jiangxi, China; 4 BGI-Shenzhen, Beishan Industrial Zone, Shenzhen, China; 5 School of Computing, Clemson University 2545, Clemson, South Carolina, USA; Corporación CorpoGen, Bogotá D.C., Colombia

**Keywords:** citrus canker, *Xanthomonas citri*, propionate, cell dormancy

## Abstract

Citrus canker, caused by *Xanthomonas citri* subsp. *citri* (Xcc), poses a substantial threat to global citrus production. Current disease control relies on the widespread application of copper-based bactericides. However, the excessive and recurrent use of copper has resulted in adverse outcomes, such as the emergence of copper-resistant strains and detrimental impacts on both the environment and human health. In this study, we demonstrate that propionate effectively inhibits the growth of *Xanthomonas citri* and is degradable by soil microbes, making it a promising and economic alternative for citrus canker management. Propionate exerts pleiotropic effects on Xcc by disrupting membrane potential, motility, and the type III secretion system. It acts as a bacteriostatic agent by acidifying the bacterial cytoplasm and disrupting proton homeostasis, which depletes intracellular ATP and induces cell dormancy. Through a Tn5 mutant library screen, we identified a propionate-sensitive mutant with a disrupted *prpC* gene, part of the *prp* operon, which is involved in propionate metabolism and regulated by PrpR. PrpR represses the *prp* operon by binding to a palindromic motif in the *prpB* promoter region. Our findings highlight propionate as a favorable control agent for citrus bacterial canker, offering effective disease management while mitigating the environmental and health concerns associated with copper-based treatments.

**IMPORTANCE:**

Citrus canker severely impacts citrus production, threatening a major fruit industry. Traditionally, managing this disease has depended on copper-based bactericides, which bring significant downsides, including copper resistance in pathogens and environmental toxicity. This study identifies propionate as a promising alternative to copper treatments for combating citrus canker caused by *Xanthomonas citri* subsp. *citri*. Propionate offers a dual benefit: it disrupts essential bacterial functions, effectively controlling the pathogen, and it is biodegradable in soil, which reduces environmental impact. Our findings show that propionate acidifies cytoplasm and disrupts critical bacterial processes such as membrane potential and motility, while depleting intracellular ATP and inducing cell dormancy. Propionate, therefore, emerges as an eco-friendly, cost-effective option for sustainable management of citrus bacterial canker, addressing both agricultural and environmental challenges in citrus production.

## INTRODUCTION

Citrus canker, caused by *Xanthomonas citri* subsp. *citri* (Xcc), is a significant disease affecting citrus production worldwide, leading to substantial economic losses (1, 2). The pathogen enters plants through natural openings or wounds, utilizing the type II-IV secretion system to transport various virulence factor proteins out of bacterial cells. These proteins attack the immune system of host plant cell, participating in the infection process (3). Infected areas on plants develop yellow, water-soaked lesions that gradually become corky and depressed, leading to symptoms such as wilting, leaf drops, and fruit loss. Infected fruits exhibit visible lesions and rot, resulting in yield and quality loss (4).

Currently, copper-based agents are widely used to control citrus canker (5). The mechanisms of copper-based treatments involve altering the protein structure, disrupting the utilization of essential elements, damaging cell membrane integrity, and affecting cell metabolism (6). Despite the widespread use of copper-based agents in agriculture, they pose potential risks and issues, including (i) accumulation of copper in the soil with long-term, excessive use, affecting the soil ecosystem and having adverse effects on soil microbiota and plants; (ii) phytotoxic effects on plants, impacting fruit quality (5); (iii) runoff or agricultural drainage leading to increased copper levels in surrounding water bodies, posing toxicity to aquatic organisms and endangering aquatic ecosystems (7); and (iv) the potential development of copper resistance in pathogenic microorganisms and pests, reducing the effectiveness of copper pesticides (8, 9). Therefore, developing new environmentally friendly control agents against Xcc is of great significance.

Propionate is a common short-chain fatty acid (SCFA) widely used in the food industry, feed additives, cosmetics, and personal care products due to its excellent antimicrobial activity (10–12). In the human intestinal tract, intestinal microbiota ferment undigested carbohydrates and dietary fiber to produce propionate as a major metabolic product, influencing the immune system and composition and function of the intestinal microbiota (13). Studies have found that *Bacteroides* spp. produces propionate metabolically to resist the colonization of *Salmonella* in the intestines (14). Increased propionate levels in the intestines are associated with alleviating pulmonary inflammation (15). Higher propionate levels in the gut also contribute to preventing excessive dietary intake and weight gain in adults (16). The antimicrobial mechanism of propionate varies among different bacteria. Sodium propionate can inhibit the synthesis of β-alanine in *Escherichia coli*, and in *Streptococcus* mutans, sodium propionate inhibits bacterial growth by altering the synthesis of alanine (17). Propionate can also inhibit biofilm formation in *Salmonella*, contributing to its antimicrobial effects (18). In *Mycobacterium tuberculosis*, propionate inhibits the transcription of *dnaA* by regulating the activity of the PrpR transcription factor, hindering the replication of chromosomal DNA (19).

Many studies have indicated that bacteria metabolize propionate through the *prp* gene cluster. In *E. coli*, the pathway of oxidizing sodium propionate to propionyl-CoA involves five enzymes: PrpBCDE and AcnB (20). Expression of the *prpBCDE* operon in *Salmonella enterica serovar* Typhimurium requires the presence of either the PrpE protein or the cellular acetyl-CoA synthesis system for propionyl-CoA synthesis and PrpC protein for 2-methylcitric acid synthesis from propionyl-CoA (21). The 2-methylcitric acid cycle (2-MCC) is a common pathway for propionate degradation metabolism in microorganisms. In *Bacillus subtilis*, when 2-methylcitric acid is present, the activator PrpR enhances the transcription of the *prpBCDE* operon. 2-Methylcitric acid is an intermediate of the 2-MCC and can convert propionate to propionyl-CoA (22).

In this study, we show that propionate efficiently inhibits the growth of *Xanthomonas citri* and can be served as a favorable control agent of citrus bacterial canker. The presence of propionate leads to pleiotropic effects on bacterial physiological processes and pathways, including defects in membrane potential, motility, and expression of type III secretion system. We reasoned that those effects are ascribed to cytosolic acidification by propionate, which acts as a bacteriostatic agent that induces bacterial dormancy. A *prp* operon regulated by PrpR was found to be involved in propionate sensitivity of Xcc via a screening of a Tn5 mutant library. Importantly, propionate can be efficiently degraded by soil microbes, implying that propionate has great potential to be an eco-friendly antimicrobial compound for controlling citrus canker.

## RESULTS

### Propionate effectively inhibits the growth of Xcc CQ13

**Fig 1:**
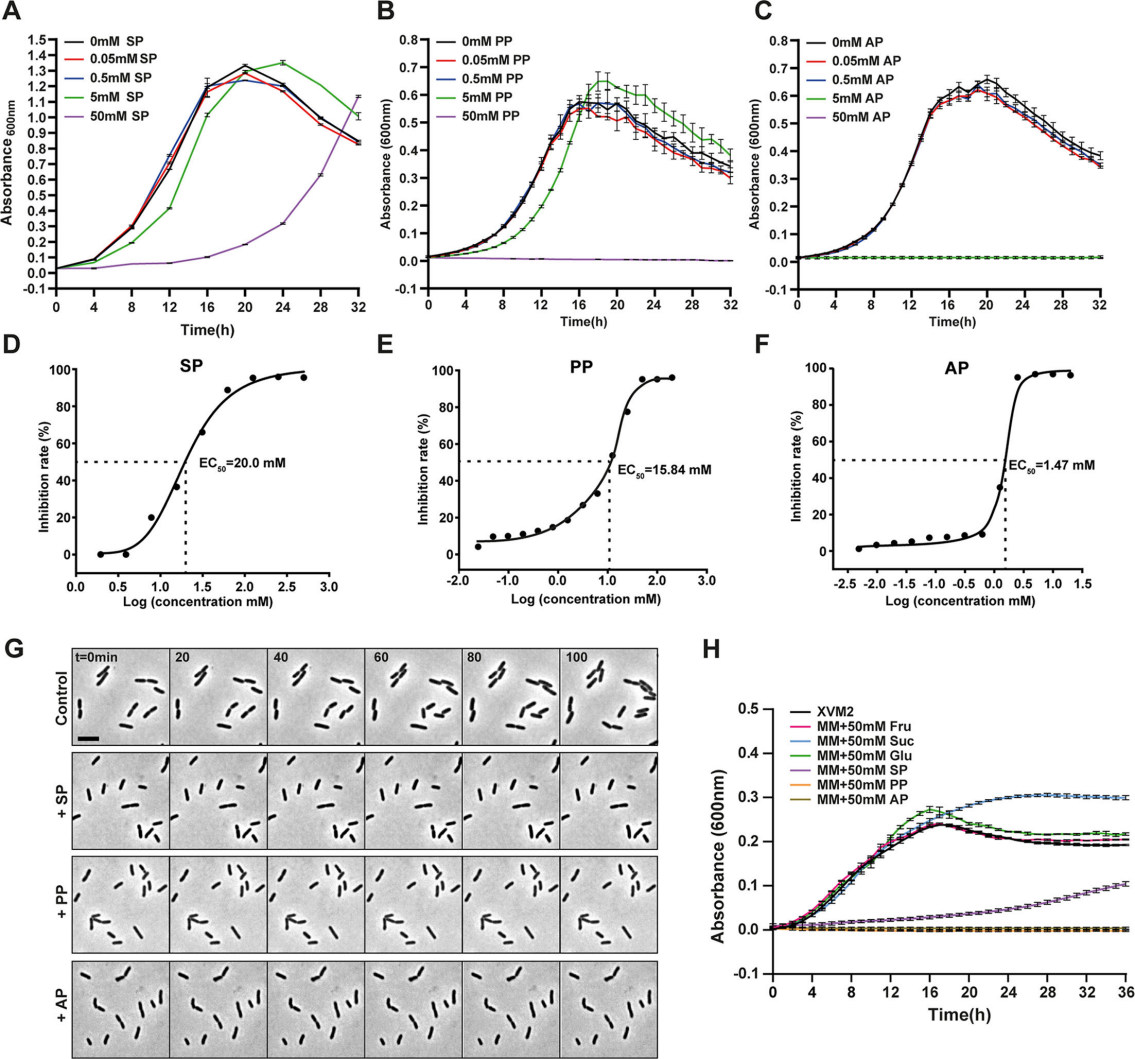
Propionate inhibits Xcc CQ13 growth. (**A–C**) Growth curves of Xcc CQ13 in nutrient broth (NB) medium supplemented with a 10-fold concentration gradient of each propionate type, ranging from 0.05 mM to 50 mM. Growth with (**A**) sodium propionate, (**B**) potassium propionate, and (**C**) ammonium propionate is shown. Cultures were grown overnight, diluted to an OD600 of 0.03, and incubated in NB medium with the specified propionate. Experiments were conducted in 24-well plates using a Tecan microplate reader. Data represent the mean and SD of at least two independent experiments with three biological replicates each (*n* = 6). (**D–F**) Dose-response curves and EC50 values for each propionate. (**G**) Time-lapse imaging of Xcc CQ13 treated with propionate. Cultures were diluted and prepared on XVM2 agar pads with or without propionate and then imaged at 28°C. Experiments were repeated more than three times, with a representative image shown. Scale bar = 2 μm. (**H**) Carbon preference analysis of Xcc CQ13. Cultures were grown overnight, diluted to an OD600 of 0.03 in XVM2 medium with different carbon sources, and measured in 24-well plates using a Tecan microplate reader. Data show mean and SD from two independent experiments, each with three biological replicates *(n* = 6). Suc, sucrose; Fru, fructose; Glu, glucose; SP, sodium propionate; PP, potassium propionate; AP, ammonium propionate.

We examined the inhibitory effects of different propionates on the growth of Xcc, including sodium propionate (SP), potassium propionate (PP), ammonium propionate (AP), and calcium propionate (CP). All tested propionates had an inhibitory effect on bacterial growth of Xcc, among which AP had the highest inhibition degree (Fig. 1A through C). Unfortunately, as CP tends to form precipitates when it interacts with other compounds in the medium, potentially undermining its antibacterial effect (Fig. S1A), we chose to focus on SP, PP, and AP in subsequent studies. To quantify the inhibitory effect of propionate on the growth of Xcc CQ13, we measured the inhibitory medium concentration (EC_50_), representing half of the maximum inhibitory concentration, which showed an EC_50_ value of 20.0 mM for SP and 15.84 mM for PP. Notably, AP showed the strongest inhibition with an EC_50_ value of 1.47 mM (Fig. 1D through F). To gain a deeper understanding of how propionate affects the growth of Xcc CQ13, we conducted time-lapse microscopy analysis of Xcc CQ13 cells grown on XVM2 agar pads. At the single-cell level, propionate extended the time to the first cell division and decreased the rate of elongation during exponential growth (Fig. 1G; Movies S1–S4).

The above results demonstrated the potent inhibitory effect of propionate on bacterial growth. Given that propionate is known to be metabolized and utilized by various microorganisms such as *E. coli*, *Salmonella*, and others (23, 24), we investigated the carbon source preference of Xcc. We observed that the growth of Xcc cells was nearly halted when propionate was supplied as the sole carbon source, whereas other sugar carbon sources supported their growth. This suggests that propionate is not the preferred carbon source for Xcc CQ13, and its antibacterial activity takes precedence over its role as a carbon source provider (Fig. 1H). In addition, we examined the inhibitory effect of propionate on the growth of two other gamma-proteobacteria, *E. coli* DH5α and *Pectobacterium brasiliense* SX309. Our data revealed that SP efficiently inhibited the growth of *P. brasiliense* SX309 (Fig. S1B), but not *E. coli* DH5α (Fig. S1B). This suggests that propionate’s effectiveness varies among different bacterial species. In summary, our findings demonstrate that propionate exhibits significant antibacterial potential for the management of citrus canker disease.

### Propionate provides a significant control and prevention effect for disease management

**Fig 2:**
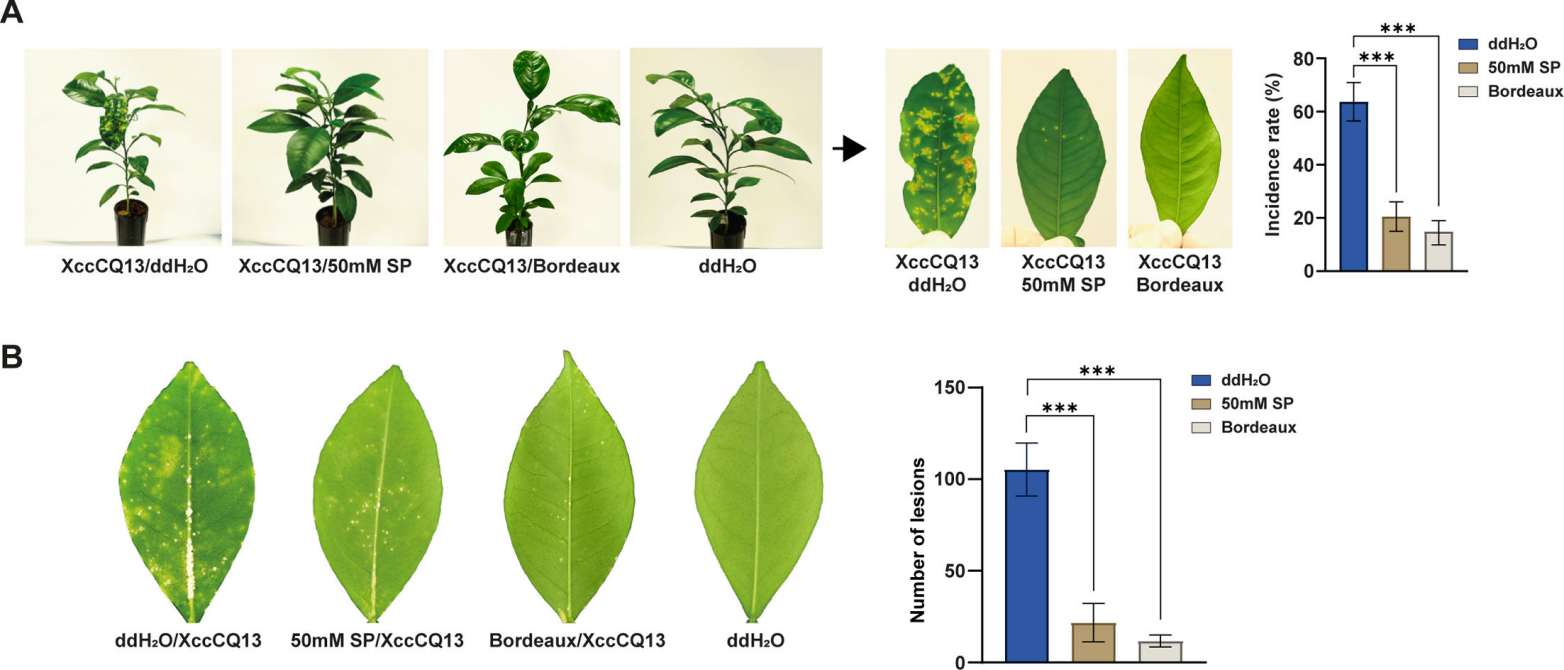
Sodium propionate effectively provides both control and prevention for citrus canker disease management. (**A**) Xcc cells (10⁸ CFU/mL) were inoculated onto grapefruit plants, followed by treatment with sodium propionate (SP, 45 mM) at 0.5, 3, and 6 days post-inoculation. Bordeaux mixture served as a positive control. Photographs were taken 25 days after inoculation. The left panel shows the overall plant condition, while the right panel presents close-ups of diseased leaves and statistical analysis of disease incidence rates. (**B**) SP (45 mM) was applied to grapefruit plants prior to Xcc inoculation (10^8^ CFU/mL). Bordeaux mixture served as a positive control. Treated leaves were incubated in a petri dish at 30°C with 90% humidity and photographed 15 days post-inoculation. Each experiment was repeated at least three times with consistent results; one representative leaf is shown. Data represent mean ± SD from two independent experiments, each including at least six leaves. Three asterisks indicate a significant difference (*P* < 0.001) based on one-way ANOVA.

To evaluate the effectiveness of propionate in controlling canker disease, we conducted spray inoculation by applying Xcc cells onto grapefruit leaves, followed by treatment with either water or SP. Bordeaux mixture served as a control. Plants treated with SP showed a remarkable reduction in disease index compared to the untreated control (Fig. 2A). Statistical analysis of incidence rates confirmed these results, with significantly lower disease incidences in SP-treated plants than in untreated ones (Fig. 2A). To assess the preventive effect of propionate, SP was sprayed before pathogen inoculation. Leaves pre-treated with SP displayed fewer symptoms than untreated leaves, as further confirmed by statistical analysis of lesion counts, which showed significantly fewer canker pustules on SP-treated leaves (Fig. 2B). Additionally, we tested the effects of potassium propionate and ammonium propionate on citrus canker control, finding their efficacy to be comparable to or even better than that of SP (Fig. S2A and B). Importantly, no phytotoxicity was observed with any propionate treatments within 30 days (Fig. S2C). These results demonstrate that propionate effectively provides both control and prevention for citrus canker disease management.

### Propionate can be efficiently degraded by soil microbes

**Fig 3:**
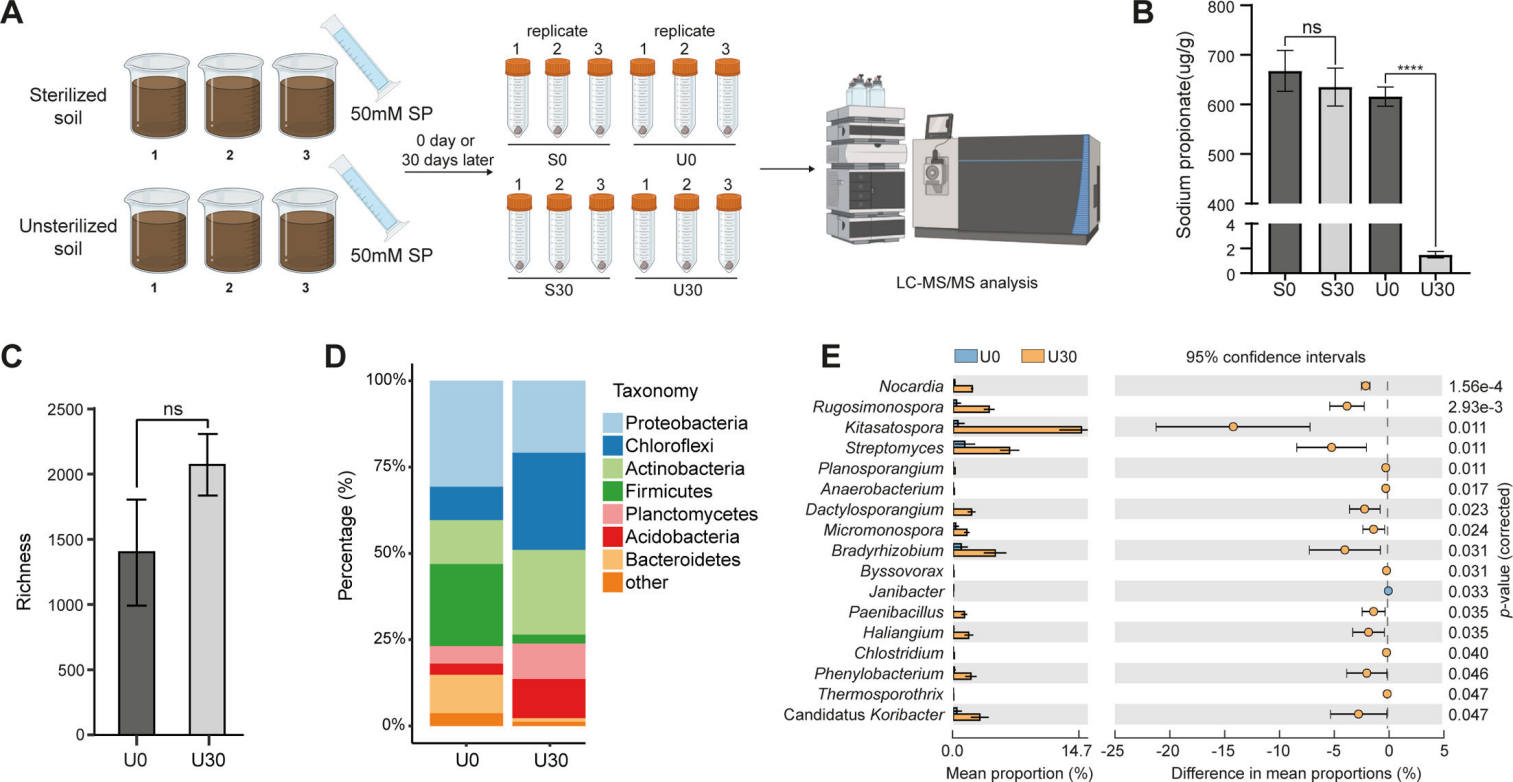
Propionate as a degradable antimicrobial compound. (**A**) LC-MS/MS workflow for targeted detection of short-chain fatty acids (SCFAs) in soil metabolome analysis. (**B**) Metabolism of sodium propionate in sterilized (left) and non-sterilized (right) soil at 0 and 30 days. Data represent means ± SD from three independent experiments; four asterisks denote a significant difference (*P* < 0.001, *t*-test). (**C**) Alpha diversity analysis of microbial community richness in sodium propionate-treated (U30) vs untreated (U0) soil, showing no significant difference (ns, *P* > 0.05, *t*-test). (**D**) Relative abundance of microbial communities at the phylum level in sodium propionate-treated (U30) and untreated (U0) soil. (**E**) Differentially abundant metagenome-assembled genomes (MAGs) between sodium propionate-treated (U30) and untreated (U0) soil. Significance was determined by Student’s *t*-test, with *P*-values corrected using the Bonferroni method.

The use of copper-based control agents leads to the accumulation of heavy metals in soil, posing risks to both soil and human health. To investigate the stability of propionate in soil, we collected soil samples from a citrus grove in Heyuan City, Guangdong Province. After inoculating the soil with 50 mM sodium propionate (SP) and incubating it for 30 days, we observed approximately 99% degradation of propionate in unsterilized soil (Fig. 3A and B). In contrast, only 5% degradation occurred in sterilized soil (Fig. 3B), and no transformation into other short-chain fatty acids was detected during the process (Fig. S3A). These results indicate that the instability of propionate is associated with soil microbiomes.

To identify the microbial structures responsible for propionate degradation, we analyzed 16S rRNA sequences. DNA was extracted from the soil and sent for library construction and sequencing. Alpha diversity analysis revealed no significant differences between the untreated (U0) and treated (U30) soil samples, indicating that propionate treatment did not dramatically alter overall soil microbial diversity (Fig. 3C). A total of 35 bacterial phyla were identified, with the most dominant phyla in U0 being Proteobacteria, Firmicutes, Actinobacteria, Bacteroidetes, and Chloroflexi (Fig. 3D). Compared to U0, U30 exhibited an increased abundance of Chloroflexi (28.10% vs 9.70%), Actinobacteria (24.53% vs 12.72%), Acidobacteria (11.33% vs 3.23%), and Planctomycetes (10.29% vs 5.08%) (Fig. 3D), suggesting that these bacterial phyla are enriched in response to propionate treatment and may be associated with its degradation. Further analysis revealed that *Lactobacillus* (6.57%), *Pseudomonas* (5.65%), and *Acidothermus* (3.87%) were the dominant genera in U0, while *Kitasatospora* (5.99%) and *Acidothermus* (5.16%) were dominant in U30 (Fig. S3B). Additionally, in U30, we observed a significant enrichment of specific bacterial communities, including *Candidatus Koribacter* (Acidobacteria), members of the *Haliangiaceae* family (Proteobacteria), and several Actinobacteria genera such as *Dactylosporangium*, *Micromonospora*, *Planosporangium*, and *Kitasatospora* (Fig. 3E). This pattern suggests a selective enrichment of specific bacterial populations following propionate treatment, with a notable increase in taxa associated with the phylum Actinobacteria, followed by Proteobacteria. Taken together, our results demonstrate that soil microbes are responsible for propionate degradation, with specific microbial communities potentially contributing to this process.

### Transcriptome analysis unveils pleiotropic effects on cellular processes upon propionate treatment

While propionate offers numerous advantages as a control agent for citrus canker, its precise antimicrobial mechanism in *Xanthomonas* remains unclear. To investigate this, we examined the transcriptional profiles of wild-type CQ13 treated with 0.5 mM sodium propionate (SP0.5) and 50 mM sodium propionate (SP50). Principal component analysis (PCA) demonstrated clear clustering of the different sample groups, with replicates within each group closely aligned, indicating the reliability and consistency of the transcriptional changes (Fig. S4A). Differentially expressed genes (DEGs) identified by RNA-seq were further validated by qPCR (Fig. S4B). In total, 58 DEGs (40 upregulated and 18 downregulated) were identified under SP0.5 treatment (Fig. S4C), while 1,434 DEGs (522 upregulated and 912 downregulated) were detected under SP50 treatment (Fig. S4D). These observations suggest that cellular responses to propionate, as reflected in gene expression patterns, are concentration-dependent.

**Fig 4:**
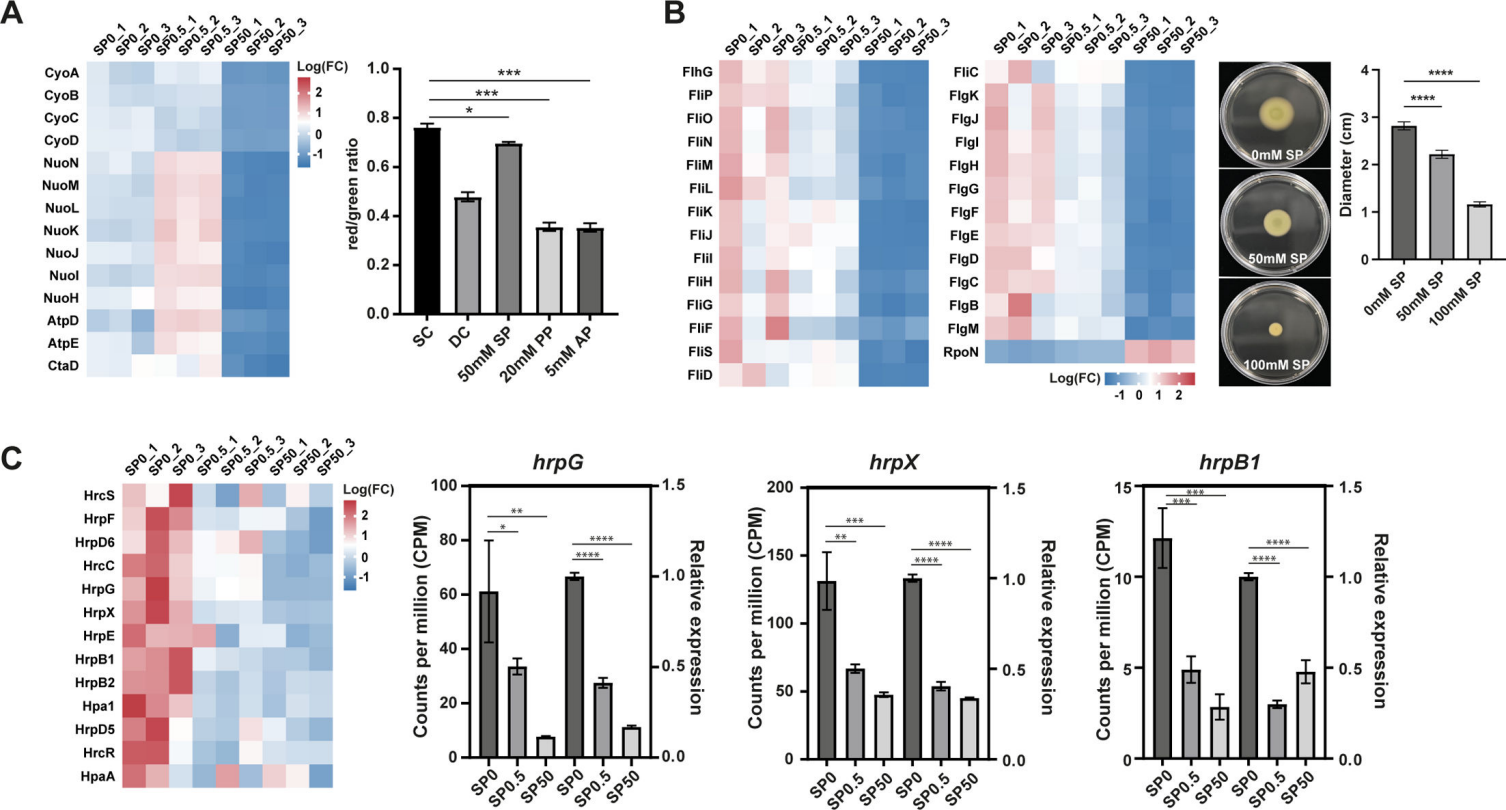
Sodium propionate inhibits flagellar assembly, oxidative phosphorylation, and type III secretion system in Xcc CQ13. (**A**) Heatmap showing gene expression profiles related to oxidative phosphorylation (left). Membrane potential assay (right) with Xcc CQ13 cells treated for 15 min with CCCP (carbonyl cyanide *m*-chlorophenyl hydrazone), sodium propionate, potassium propionate, or ammonium propionate. Cells were stained for 30 min with 30 μM DiOC₂ (3) dye. The bar plot shows the red/green fluorescence ratio indicating membrane potential. Mean ± SD from three independent biological replicates. Asterisks indicate a significant difference (*P* < 0.05) and three asterisks indicate *P* < 0.001 by *t*-test. SC, stain control; DC, depolarized control. (**B**) Heatmap showing gene expression profiles related to flagellar assembly (left). Motility assay on 0.28% nutrient agar plates (right) after 72 h of incubation. One representative result is shown from three independent experiments. Mean ± SD from three independent biological replicates (*P* < 0.0001 by *t*-test). (**C**) Heatmap showing gene expression profiles of type III secretion system (T3SS) genes (left). qRT-PCR analysis (right) of representative T3SS regulators and genes (*hrpG*, *hrpX*, and *hrpB1*) in response to propionate treatment. Four asterisks indicate *P* < 0.0001 by *t*-test.

All DEGs resulting from propionate treatment were subjected to KEGG pathway enrichment analysis. Under SP0.5 treatment, DEGs were significantly enriched in six metabolic pathways (*q*-value < 0.05), including propionate metabolism (Fig. S4F). In contrast, under SP50 treatment, pathways related to ribosome, two-component systems, flagellar assembly, and bacterial chemotaxis were enriched (*q*-value < 0.05) (Fig. S4G). The expression patterns of DEGs involved in key metabolic pathways are presented as heatmaps in Fig. 4 and Fig. S4E.

Notably, several of the most downregulated genes were involved in oxidative phosphorylation, the final stage of cellular respiration, which is crucial for providing energy for cellular activities. To verify whether oxidative phosphorylation in Xcc CQ13 was inhibited by SP, we assessed the membrane potential of bacteria treated with 50 mM SP using the membrane potential-sensitive dye DiOC2(3) and flow cytometry. The results showed a significant decrease in membrane potential upon treatment (Fig. 4A). Additionally, we examined the effects of PP and AP on cell membrane potential and found that these propionates reduced membrane potential to a greater extent than SP (Fig. 4A). Furthermore, genes related to motility and the type III secretion system (T3SS) were found to be downregulated, potentially explaining the inhibitory effect of propionate on bacterial pathogenesis. Bacterial swimming was significantly inhibited on semi-solid NA plates treated with either 50 mM or 100 mM SP (Fig. 4B). The expression levels of three representative T3SS genes (*hrpG*, *hrpX*, and *hrpB1*) were assessed by qRT-PCR, and the results showed that the trend in gene expression changes was consistent with the RNA-seq data, indicating that propionate can inhibit the expression of the bacterial type III secretion system (Fig. 4C).

### Propionate induces bacterial dormant-like state by acidifying bacterial cytoplasm and depleting intracellular ATP

**Fig 5:**
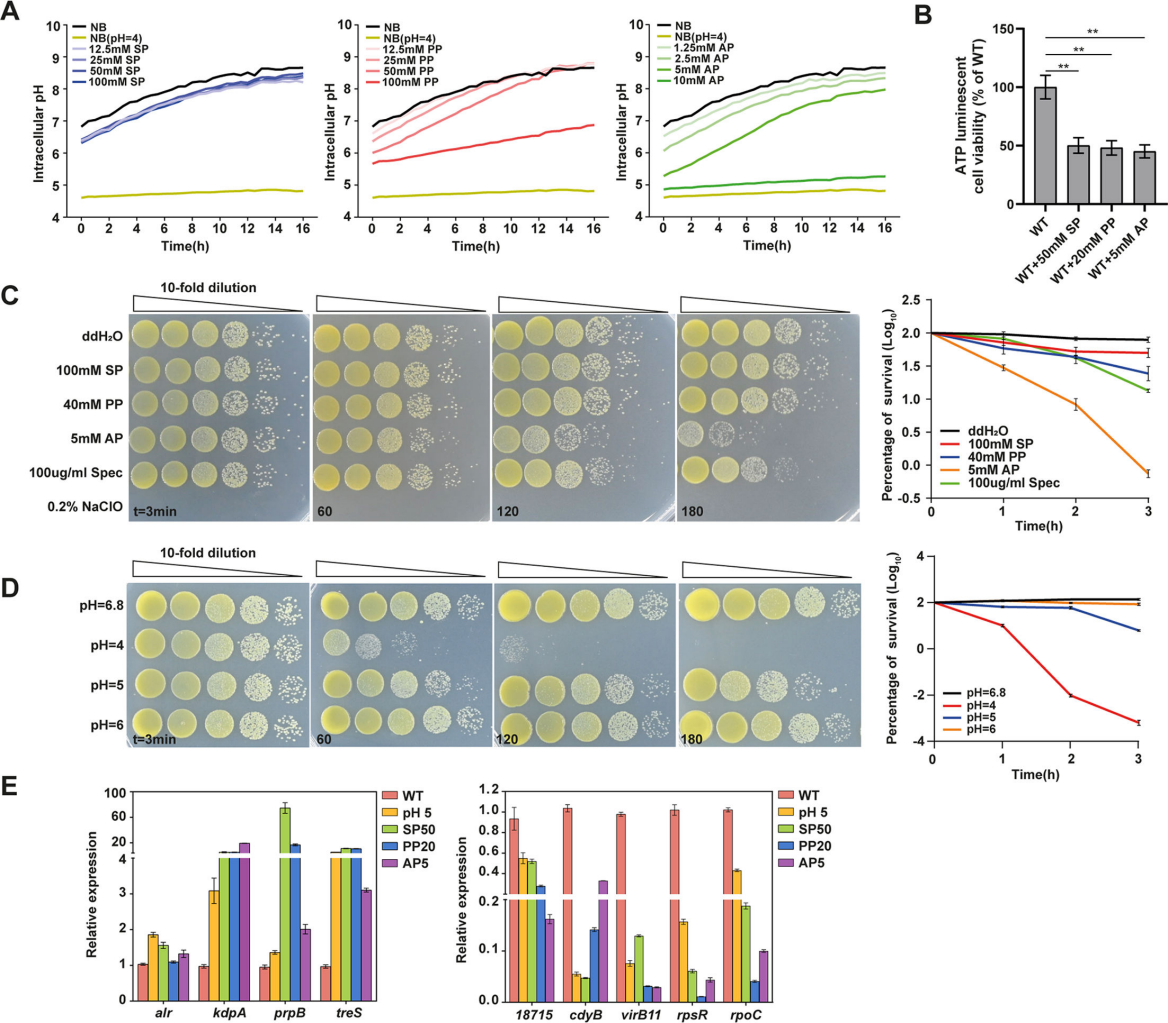
Propionate induces cell dormancy via cytoplasmic acidification and intracellular ATP depletion in Xcc CQ13. (**A**) Xcc CQ13 cells were cultured to OD600 = 1 and transferred to fresh NB medium with varying pH (4–6). BCECF-AM (2 μM) and CCCP (50 µM) were added for fluorescence ratiometric pH measurements at λ_em_ = 488 nm and λ_em_ = 440 nm under λ_ex_ = 535 nm using a plate reader (Tecan). A ratio-pH standard curve was generated for absolute intracellular pH determination. Propionate treatment was initiated for 15 min, followed by BCECF-AM addition, and fluorescence intensity was monitored for 16 h. Data represent three independent biological duplicates. (**B**) Wild-type Xcc CQ13 was treated with sodium propionate (SP, 50 mM), potassium propionate (PP, 20 mM), and ammonium propionate (AP, 5 mM). ATP luminescence was measured to assess viability (mean ± SD, *n* = 5). Significance is denoted by two asterisks (*P* < 0.001) using a *t*-test. (**C**) Xcc CQ13 cells were cultured to OD600 = 0.5 and exposed to various stressors including 0.2% NaClO, 100 µg/mL spectinomycin, and propionates (0.2% NaClO as a negative control, ddH_2_O as a positive control, and spectinomycin as a bacteriostatic control) for 3 min, 1 h, 2 h, and 3 h. Survival was assessed by CFU counting after 10-fold dilution on NA plates. Results are expressed as percent survival relative to untreated cells (mean ± SD, *n* = 6 from at least two independent experiments). (**D**) Cells were cultured to OD600 = 0.5 and incubated in NB medium adjusted to pH 4–6 for 3 min, 1 h, 2 h, and 3 h. Survival was determined by CFU counting on NA plates after 10-fold dilution. pH 6.8 represents the unadjusted pH of NB medium. Survival is expressed as percent survival relative to untreated cells (mean ± SD, *n* = 6 from at least two independent experiments). (**E**) Relative gene expression levels were assessed in acidified NB (pH 5) and NB with propionate. Genes were selected from the RNA-seq data set for their representativeness in differential expression. Two independent experiments were conducted with three biological replicates each (*n* = 6). Treatments include acidic NB (pH 5), SP50 (50 mM sodium propionate), PP20 (20 mM potassium propionate), and AP5 (5 mM ammonium propionate).

The compromised membrane potential observed upon SP treatment could be attributed to a disrupted intracellular-to-extracellular proton gradient, known as the proton motive force (PMF). Propionate can diffuse into the bacterial cytoplasm and ionize to form propionic acid, while cations are expelled by Na^+^/K^+^ transporters or efflux pumps. This process leads to cytoplasmic acidification, which negatively impacts protein folding and enzyme activities (25, 26). To test whether propionate treatment causes cytoplasmic acidification, we measured intracellular pH during bacterial growth in the presence of propionate using the cell-permeable pH-sensitive dye BCECF-AM. We found that Xcc CQ13 grown in NB medium rapidly buffered its cytoplasm to a more neutral pH during growth (Fig. 5A), consistent with previous studies of intracellular pH during aerobic growth (27). In contrast, cells grown in propionate exhibited significantly slower cytoplasmic buffering, with those grown in AP showing the slowest buffering rate (Fig. 5A). These data suggest that intracellular pH buffering is significantly slowed during propionate treatment, likely contributing to the prolonged lag phase and reduced growth rate.

The acidification of the bacterial cytoplasm, coupled with membrane potential dysfunction, may lead to instability and insufficient synthesis of intracellular ATP. We confirmed that intracellular ATP levels decreased by 50% following propionate treatment (Fig. 5B). It has been proposed that low intracellular ATP levels are a primary driver of bacterial cell dormancy (28). To investigate this, we assessed the survival rate of Xcc cells after exposure to propionate for varying durations. We observed that cell growth resumed after propionate treatment although the survival rate was lower when treated with AP (Fig. 5C). The effect of propionate treatment was comparable to that of spectinomycin, a typical bacteriostatic antibiotic, suggesting that propionate acts as a bacteriostatic agent capable of blocking Xcc cell growth (Fig. 5C). To determine whether this growth inhibition is associated with cytoplasmic acidification, we conducted similar survival rate experiments under acidic stress. Cells underwent growth arrest in acidic medium but could recover similarly to those treated with propionate although with a lower recovery rate at pH 4 (Fig. 5D). No decreased bacterial survival rate was observed in alkaline medium (Fig. S5). Additionally, nine propionate-induced DEGs identified in the RNA-seq analysis exhibited similar expression changes when cells were grown in acidic medium (Fig. 5E). Collectively, these results demonstrate that propionate protonation acidifies the bacterial cytoplasm and depletes intracellular ATP, leading to transient growth inhibition in Xcc. Given that ATP depletion and growth arrest are hallmarks of bacterial dormancy, our findings suggest that propionate treatment induces a dormant-like state in Xcc.

### PrpC in *prp* operon plays critical role in regulating propionate sensitivity and metabolism in Xcc CQ13

**Fig 6:**
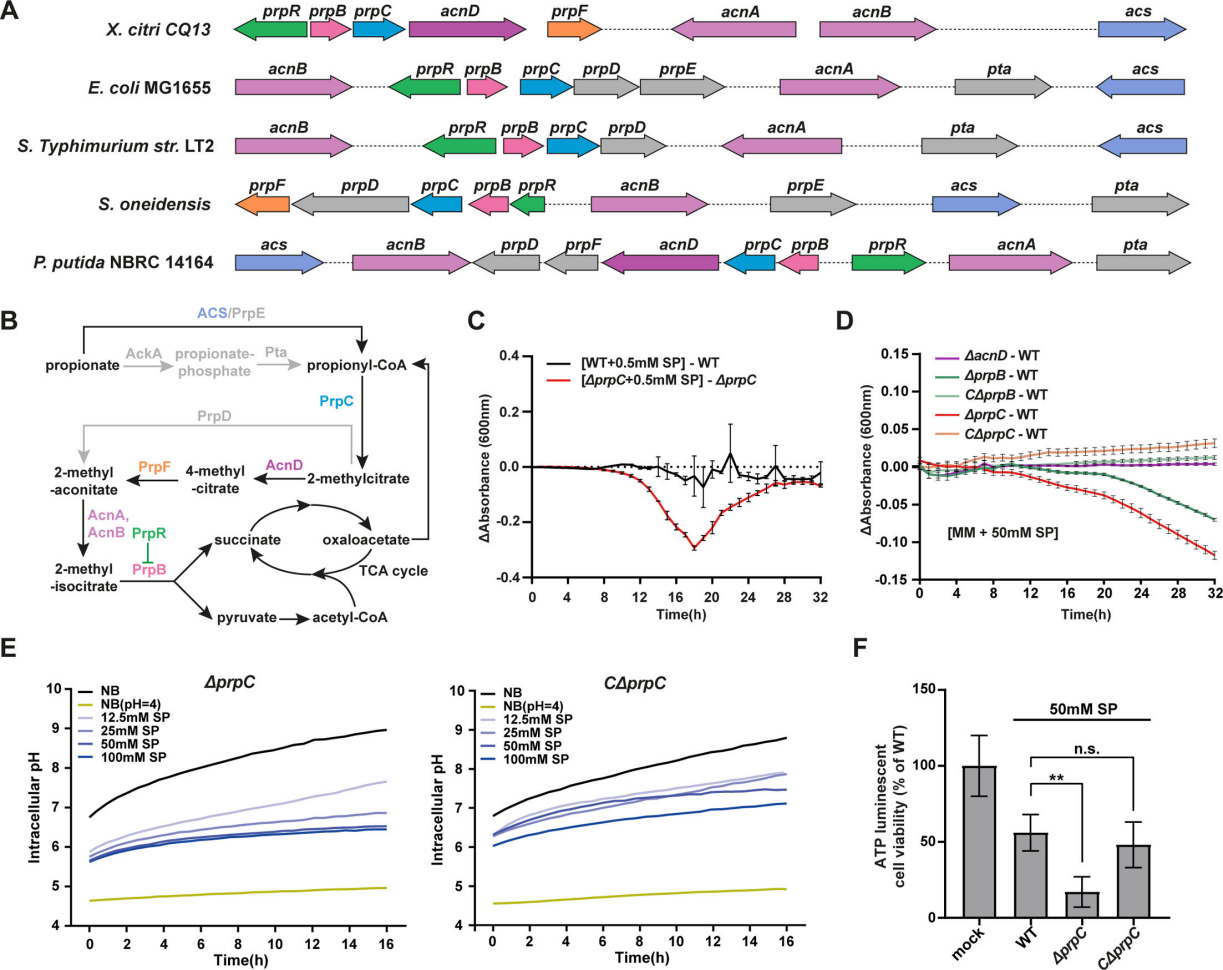
PrpC plays a critical role in propionate metabolism. (**A**) Organization of propionate metabolism genes across *X. citri* CQ13, *E. coli* MG1655, *S. Typhimurium* str. LT2, *S. oneidensis*, and *P. putida* NBRC 14164. (**B**) Metabolic pathway of propionate in Xcc and other bacteria. Black lines represent Xcc pathways, while gray lines indicate pathways present in other bacteria but absent in Xcc. Proteins involved in the pathway are color-coded to match the gene organization in panel **A**. (**C**) Sensitivity of the *prpC* mutant to 0.5 mM sodium propionate. The delta absorbance represents the difference in absorbance values between strains cultured in NB medium with 0.5 mM sodium propionate and those in standard NB medium. (**D**) The delta absorbance represents reduced propionate metabolism capabilities of *prpB* and *prpC* deletion mutants using SP as a sole carbon source in MM medium, while the *acnD* mutant exhibits no significant change. The complementation strains of *prpB* and *prpC* mutants show restored phenotypes. Mean ± SD from at least two independent experiments with three biological replicates (*n* = 6) are presented. (**E**) Intracellular pH of Δ*prpC* and complementation strain. Cells were cultured to OD600 = 1 and transferred to fresh NB medium with 50 mM SP. BCECF-AM (2 μM) and CCCP (50 μM) were added for fluorescence ratiometric pH measurements at λ_em_ = 488 nm and λ_em_ = 440 nm under λ_ex_ = 535 nm. A ratio-pH standard curve was generated for absolute intracellular pH determination. Propionate treatment was initiated for 15 min, followed by BCECF-AM addition, and fluorescence intensity was monitored for 16 h. Data represent three independent biological duplicates. (**F**) Intracellular ATP assay. Strains were treated with 50 mM SP. ATP luminescence was measured to assess viability (Mean ± SD, *n* = 5). Significance is denoted by two asterisks (*P* < 0.001) using a *t*-test.

To further investigate how Xcc cells respond to propionate stress, we screened a hyper-saturated Tn5 mutant library previously established in our lab (29). Despite multiple attempts, we were unable to isolate any mutants resistant to propionate treatment, including spontaneous mutants by directly plating bacterial cells on propionate-supplemented plates. However, we successfully isolated mutants that exhibited increased sensitivity to propionate. The Tn5 insertion sites were mapped using plasmid rescue and were found within the coding region of the *prpC* gene, part of the *prp* operon (which includes *prpB*, *prpC*, *acnD*, and *prpF*), responsible for regulating propionate metabolism. Comparative analysis of this operon in Xcc and other bacteria (Fig. 6A) revealed that Xcc lacks the PrpE and AckA/Pta-mediated propionyl-CoA pathway (Fig. 6B).

To confirm the roles of *prp* operon in propionate sensitivity, we generated deletion mutants of *prpB*, *prpC*, and *acnD* through a double homologous recombination approach. However, we were unable to obtain a *prpF* mutant, likely due to its essential role in bacterial survival, which aligns with our previous findings on essential genes in *Xanthomonas citri* (29). Compared to the wild-type strain, the *prpC* mutant exhibited a significantly delayed log-phase growth in medium supplemented with 0.5 mM SP (Fig. 6C), while the *prpB* and *acnD* mutants showed no significant difference in growth compared to the wild type (Fig. S6A and B). Pathogenicity assays produced consistent results: the *prpC* mutant displayed slightly reduced virulence compared to the wild type at 4 days post-inoculation although it approached wild-type levels by 8 days post-inoculation. Meanwhile, the virulence of the *prpR*, *prpB*, and *acnD* mutants showed no significant difference compared to the wild type (Fig. S6C). To further confirm whether the *prp* operon is involved in propionate metabolism, we assayed the metabolic capability of *prp* mutants to utilize SP as the sole carbon source in minimal medium. Deletion of *prpC* completely abolished the ability to utilize SP as a carbon source for growth, while *prpB* and *acnD* mutants were still able to metabolize propionate (Fig. 6D). All *prp* mutants displayed similar growth patterns in XVM2 medium, where sugars were used as the carbon source (Fig. S6D). These data suggest that PrpC is critical for the upstream reactions in propionate metabolism, while 2-methyl-isocitrate may be metabolized through alternative pathways independent of PrpB-mediated conversion. This notion is further validated by intracellular pH and ATP assays. The *prpC* mutant exhibited lower intracellular pH value and less ability of pH buffering compared to complementary strain (Fig. 6E). Similarly, the intracellular ATP level was significantly decreased compared to that of wild type and complementary strains in the presence of propionate (Fig. 6F). These defects were not observed for *prpB* mutant (Fig. S6E and F). Given that propionyl-CoA is toxic to bacteria, as observed in *Mycobacterium tuberculosis* (30)*,* we conclude that the sensitivity of the *prpC* mutant to propionate is due to the essential role of PrpC as an upstream enzyme in the propionate metabolic pathway. The absence of PrpC likely disrupts the conversion of propionyl-CoA to 2-methylcitrate, resulting in the accumulation of toxic propionyl-CoA and inhibiting bacterial growth.

### PrpR is a transcriptional repressor that negatively regulates *prp* gene cluster

**Fig 7:**
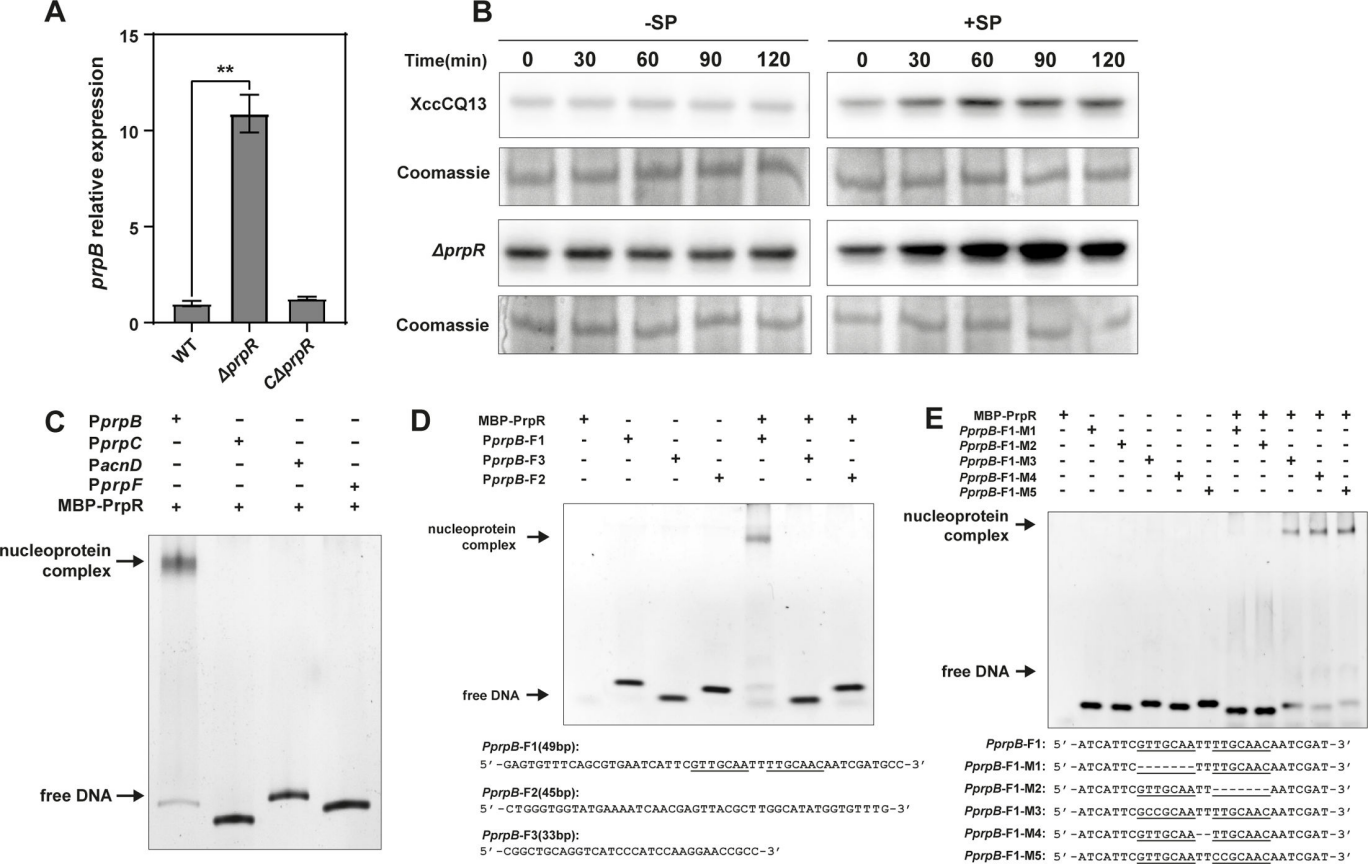
PrpR negatively regulates *prp* gene cluster. (**A**) *prp* gene expression levels in Xcc CQ13 wild-type strain (WT), *prpR* mutant strain, and *prpR* mutant complementation strain. Total RNA was extracted from cultures grown in NB medium, and mRNA levels of *prpB* were normalized to the housekeeping gene *gyrB*. Means were calculated from three independent experiments with three replicates per experiment. Two asterisks indicate a significant difference (*P* < 0.01) by *t*-test. (**B**) The promoter activity of *prpB* assayed by immunoblots in the presence and absence of the propionate. Plasmid pL2-PprpB-mCherry-Flag was introduced into the WT strain and the *prpR* mutant. mCherry protein levels were detected via immunoblotting in cultures grown in NB medium with or without sodium propionate. Coomassie staining of the blots was used as a loading control. The experiment was repeated three times with consistent results, and one representative blot is shown. (**C**) Gel shift assay for interaction between MBP-PrpR protein and *prp* promoter regions. Four DNA fragments containing the *prpB*, *prpC*, *acnD*, and *prpF* promoter regions, respectively, were incubated with 2 μM PrpR, and nucleoprotein complexes were analyzed on a 12% acrylamide gel. (**D**) Gel shift assay for MBP-PrpR interaction with the first 49 bp of the *prpB* promoter region. The *prpB* promoter was divided into three fragments: F1 (49 bp), F2 (45 bp), and F3 (33 bp). Each fragment was incubated with 2 μM MBP-PrpR, and nucleoprotein complexes were analyzed on a 12% acrylamide gel. (**E**) Gel shift assay for MBP-PrpR interaction with the palindromic sequence in the *prpB* promoter region. Mutations were introduced into the palindromic sequence within the F1 fragment through base deletions or insertions. Each mutant fragment was incubated with 2 μM MBP-PrpR, and nucleoprotein complexes were analyzed on a 12% acrylamide gel.

A *prpR* gene was identified upstream of *prpB*, suggesting it may act as a cognate regulator of the *prp* operon. qPCR results revealed that the expression level of *prpB* in the *prpR* mutant was significantly higher than in both wild-type strains and the complementary strain, indicating that PrpR negatively regulates *prpB* expression (Fig. 7A). These findings contrast with previous reports in *Salmonella enterica* (31) and *Mycobacterium tuberculosis* (32), where PrpR functions as a transcriptional activator. To further validate these results, we constructed a reporter system in which the expression of the mCherry protein is driven by the *prp* promoter. Immunoblots detected with anti-mCherry antibodies showed that the reporter protein level in the *prpR* mutant was significantly higher than in the wild-type strain. We also assessed mCherry induction in the presence of propionate and intriguingly found that mCherry protein expression was induced in both the wild type and *prpR* mutant (Fig. 7B). This indicates that induced expression of *prp* operon is propionate-dependent but not solely regulated by PrpR. While deletion of *prpR* impacts mCherry production, the fact that induction occurs in both the wild-type and *prpR* mutant strains suggests the involvement of additional regulatory factors beyond PrpR.

This observation prompted us to investigate the PrpR binding site on the *prp* promoter. The recombinant MBP-PrpR fusion protein was expressed and purified via affinity chromatography (Fig. S7A). We then amplified the upstream sequences of the *prpB*, *prpC*, *acnD*, and *prpF* genes by PCR, combined the PCR products with MBP-PrpR protein, and analyzed the protein-DNA binding using a gel shift assay. MBP-PrpR specifically bound to the upstream sequence of *prpB* (Fig. 7C), supporting our prediction that *prpB* is the first structural gene in the *prp* operon and that its upstream region likely contains the PrpR binding sequence. To precisely map the PrpR binding site, we divided the *prpB* promoter sequence into three segments and found that PrpR binds to the first 49 bp of the *prpB* promoter (Fig. 7D). Within this segment, we identified a palindromic sequence (GTTGCAA-N_2_-TTGCAAC) and speculated that this motif could be the putative PrpR binding site. To test this hypothesis, we assayed the DNA binding of MBP-PrpR with five mutated probes: deleting the first half (M1) or second half (M2) of the palindromic sequence, replacing two bases in the first half (M3), and deleting (M4) or adding (M5) two bases in the middle of the palindromic sequence. The results showed that MBP-PrpR failed to bind to M1, M2, and M3 (Fig. 7E), while M4 and M5 retained PrpR-binding ability, demonstrating that the palindromic sequence is essential for PrpR binding. We also tested three different ligands (2-methylcitrate, 2-methylcitric acid, and sodium propionate) that might allosterically regulate PrpR DNA-binding activity; however, we did not observe any changes in PrpR binding to the probe in the presence of these propionate variants, implying that PrpR may not efficiently respond to propionate or regulated by an unknown mechanism (Fig. S7B and C). These data are consistent with our previous observation that the propionate induction of the *prp* operon is independent of PrpR. Together, our findings suggest that PrpR acts as a transcriptional repressor that negatively regulates the expression of the *prp* operon by binding to a palindromic motif in the promoter region of *prpB*.

## MATERIALS AND METHODS

### Bacterial strains, growth conditions, primers, and plasmids

The bacterial strains and plasmids used in this study are listed in Table S1. The primers used in this study are listed in Table S2. The Xcc CQ13 wild-type strain and mutants were cultured at 28°C in NB (Difco Laboratories, Detroit MI), or in XVM2 medium (33), on nutrient agar (NA) plates. The MM medium used in this study is composed of XVM2 salts without any carbon source. The *E. coli* strains were grown in LB medium at 37°C. When required, antibiotics were used at the following concentrations: kanamycin (Kan) at 50 µg/mL, ampicillin (Amp) at 100 µg/mL, gentamicin (Gm) at 5 µg/mL, and spectinomycin (Spec) at 50 µg/mL.

Detailed experimental procedures, including additional methods and protocols, are provided in Text S1.

## DISCUSSION

**Fig 8:**
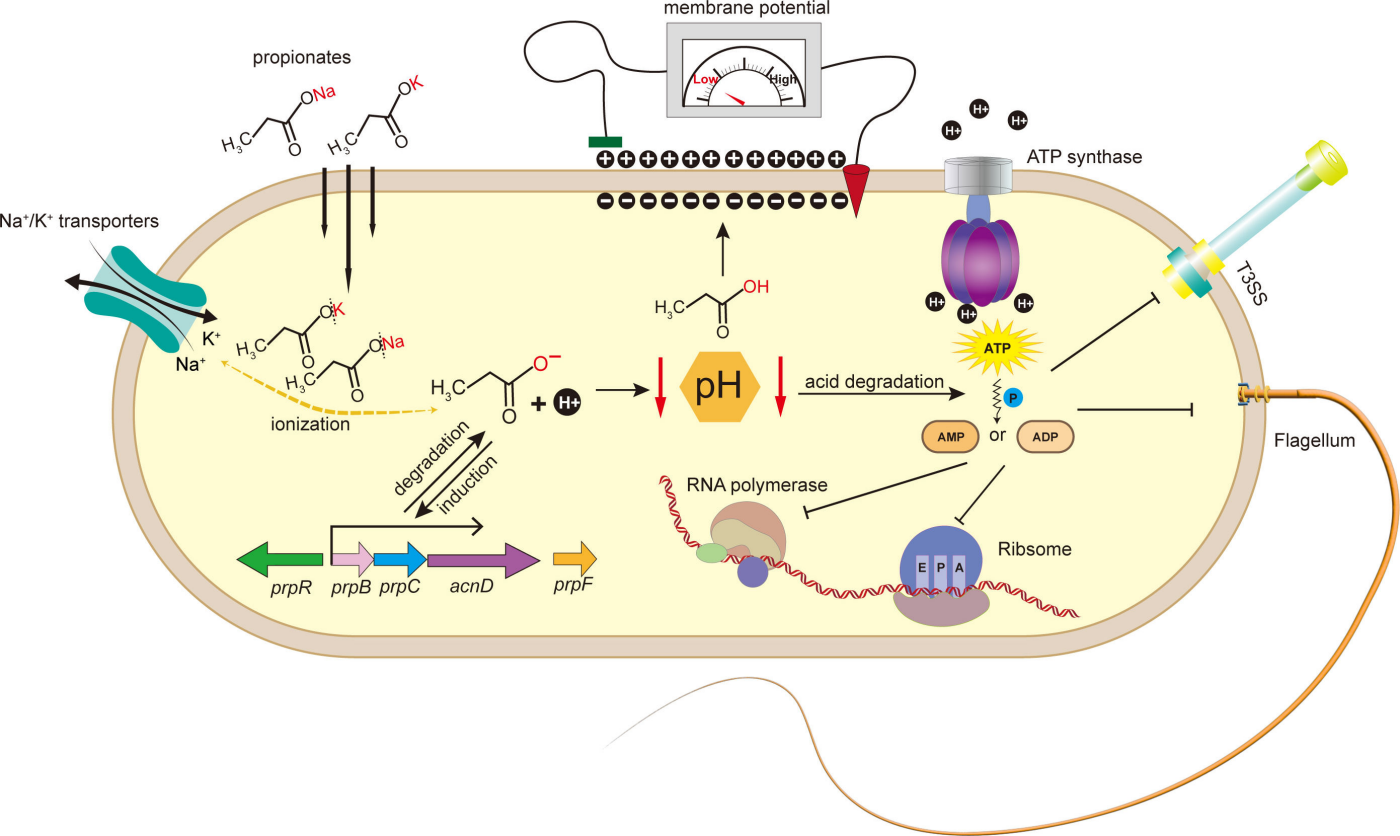
Proposed model of propionate inhibiting the growth of *Xanthomonas citri* subsp. *citri*. The diagram illustrates how propionate diffuses into the bacterial cytoplasm, where it ionizes to form propionic acid. Concurrently, cations (Na^+^/K^+^) are expelled by transporters or efflux pumps. The accumulation of propionic acid leads to cytoplasmic acidification, impairing protein folding and enzyme activity. This acidification disrupts membrane potential, causing a significant pH difference between the cytoplasm and the outer membrane, which hinders ATP synthesis and accelerates its breakdown into AMP. The depletion of ATP inhibits key cellular processes, such as bacterial motility, the type III secretion system, transcription, and translation, ultimately leading to cell dormancy. Additionally, propionate activates the expression of the *prp* operon, which modulates its metabolic degradation within *Xanthomonas citri*.

Our study proposes the use of propionate as a degradable control agent for managing citrus canker. We found that propionate effectively inhibits the growth of *Xanthomonas citri* by acidifying the bacterial cytoplasm and depleting intracellular ATP, inducing cell dormancy (Fig. 8). Importantly, propionate is degradable by soil microbes, offering an environmentally friendly strategy for citrus canker management. Additionally, we identified the regulatory mechanism of the *prp* operon involved in propionate metabolism, providing new insights into how *Xanthomonas citri* responds to propionate.

There are several potential advantages for the widespread application of propionate in the field. The bulk price of propionate is approximately 2 U.S. dollars per kilogram, making it significantly more cost-effective than current fungicides. Moreover, propionate and its salts are widely used as food and animal feed additives, posing minimal safety concerns for humans and animals. Propionate could also be used in combination with other fungicides. Additionally, metal cations (K^+^, Na^+^, Mg²^+^, Ca²^+^) combined with propionate can be absorbed by plants as essential nutrients after propionate degradation. The ammonium propionate (AP) and potassium propionate (PP) tested in this study are capable of providing nitrogen and potassium, respectively. Thus, propionate functions not only as a bacteriostatic agent, effectively inhibiting the growth of pathogenic microorganisms, but it also has potential utility as a fertilizer in agricultural applications.

Propionic acid can bind with various metal cations to form corresponding propionate complexes. This study has revealed that different types of propionate are all effective in inhibiting the growth of Xcc CQ13, yet their antibacterial efficacies exhibit notable differences (Fig. 1). Specifically, AP and PP exhibit superior antibacterial performance compared to SP. After eliminating the influence of propionic acid as the common anion, we hypothesize that the distinct cations may be the critical factor contributing to the varying antibacterial effects. Ammonium exists in different forms depending on the pH value. Through measuring the cytosolic pH, our study found that bacterial cells treated with ammonium propionate experienced the most significant decrease in intracellular pH, accompanied by the slowest buffering recovery rate, indicating that ammonium ions primarily exist in the form of NH_4_^+^ and interact with negatively charged bacterial components, disrupting cell wall integrity, enhancing cell membrane permeability, and ultimately reducing cellular bioactivity due to osmotic potential imbalance (34, 35). Similarly, the concentration gradient of potassium ions across the cell membrane is a crucial factor in maintaining osmotic pressure balance (36). The introduction of high concentrations of potassium ions disrupts this balance, perturbs the intracellular and extracellular ion concentration gradient, affects the material transport processes, and consequently diminishes cellular activity.

Propionate’s ability to inhibit bacterial growth likely varies depending on the specific bacterium. For example, sodium propionate inhibits the synthesis of β-alanine in *Escherichia coli*, and the addition of exogenous β-alanine can alleviate this inhibitory effect (37). However, this alleviation does not occur in other organisms such as *Aspergillus*, *Bacillus subtilis*, *Pseudomonas*, or *Trichophyton* (37). Our previous experiments also confirmed that the exogenous addition of β-alanine and D-alanine could not alleviate the inhibitory effects of sodium propionate on *Xanthomonas citri* (data not shown). In *Streptococcus* spp., sodium propionate impedes bacterial growth by altering methionine synthesis (17). while in *Salmonella*, it inhibits biofilm formation (18). Additionally, in *Mycobacterium tuberculosis*, propionates inhibit *dnaA* transcription, which hinders chromosomal DNA replication by regulating the PrpR transcription factor (19). We propose that the primary mechanism by which propionate inhibits bacterial growth in *Xanthomonas citri* involves disrupting the proton gradient, which is essential for generating membrane potential—a crucial source of energy for cellular activities. Membrane potential plays a key role in bacterial cell division by affecting the localization of several cell division-related proteins (38). Furthermore, our data demonstrated that propionate-induced cytoplasmic acidification leads to a depletion of intracellular ATP (Fig. 5), which may explain why genetic screening for propionate-resistant mutants was unsuccessful. This suggests that it may be more challenging to generate propionate-resistant strains compared to those resistant to copper-based pesticides.

In our study, we provided compelling evidence that PrpR functions as a transcriptional repressor, inhibiting the expression of the *prp* operon. This finding contradicts previous reports that identified PrpR as a transcriptional activator, particularly through its binding to 2-methylcitrate (31). Our experiments confirmed that PrpR in Xcc was unresponsive to 2-methylcitrate (Fig. S7). This discrepancy could be explained by the low sequence identity-Xcc PrpR shares only 45% similarity with that of *Salmonella*. Furthermore, the critical residue A162, essential for 2-MC sensing, is not conserved in Xcc CQ13. These differences align with our observation that Xcc cells are not efficient at utilizing propionate as a sole carbon source. Thus, it is likely that Xcc responds to propionate through a mechanism distinct from those previously reported. Further experiments are necessary to elucidate how the *prp* operon is induced and to determine whether additional accessory factors are involved in this regulatory process.

## Data Availability

The RNA-seq data generated in this study have been deposited in the Genome Sequence Archive (https://ngdc.cncb.ac.cn/gsa/) and are accessible under the accession number CRA017482. Additional data supporting the findings of this study are available from the corresponding author upon reasonable request.

## SUPPLEMENTAL MATERIAL

Text S1 mbio.00642-25-s0001.docx Additional experimental details.

Supplemental figures mbio.00642-25-s0002.pdf Fig. S1 to S8.

Legends mbio.00642-25-s0003.docx Supplemental figure and movie legends.

Table S1 mbio.00642-25-s0004.docx Strains and plasmids.

Table S2 mbio.00642-25-s0005.docx Primers.

Movie S1 mbio.00642-25-s0006.avi Xcc CQ13 wild-type strain grown in the XVM2 agarose pad.

Movie S2 mbio.00642-25-s0007.avi Xcc CQ13 wild-type strain grown in the XVM2 agarose pad supplemented with 50 mM SP.

Movie S3 mbio.00642-25-s0008.avi Xcc CQ13 wild-type strain grown in the XVM2 agarose pad supplemented with 20 mM PP.

Movie S4 mbio.00642-25-s0009.avi Xcc CQ13 wild-type strain grown in the XVM2 agarose pad supplemented with 5 mM AP.

ASM does not own the copyrights to Supplemental Material that may be linked to, or accessed through, an article. The authors have granted ASM a non-exclusive, world-wide license to publish the Supplemental Material files. Please contact the corresponding author directly for reuse.
