# Supplementary material for: Propionate serves as a degradable control agent of citrus canker by acidifying cytoplasm and depleting intracellular ATP in Xanthomonas citri
Source: mBio. 2025 Apr 29;16(6):e00642-25. doi: 10.1128/mbio.00642-25 (PMC12153269; doi:10.1128/mbio.00642-25)
Supplement: Text S1 — Additional experimental details. [file mbio.00642-25-s0001.docx]

# **Supplemental Materials and Methods**

**In Vitro Growth Curve Experiment**

Growth measurements were conducted using a Tecan microplate reader, with absorbance (OD 600) recorded every 15 minutes for a total of 32 hours at 28°C. All growth curve experiments in 24-well plates began with an initial OD of 0.03, with each well containing a 1 ml culture volume. Growth curve data were analyzed using GraphPad Prism 8.

**Time-Lapse Microscopy Assay**

Xcc CQ13 was grown overnight prior to spotting on either an XVM2 agar pad as a control or a pad supplemented with 50 mM sodium propionate or 20 mM potassium propionate or 2.5 mM ammonium propionate. Cells were imaged every 20 minutes for 160 minutes at 28°C. Phase-contrast images were acquired with a Leica inverted microscope (Leica DMi8) using a 100X (NA 1.40) oil-immersion objective. Processing of images was performed using ImageJ.

**16S rRNA Sequencing for Soil Microbiome**

DNA was extracted using the DNeasy PowerSoil Pro Kit (Qiagen, Hilden, Germany), and samples were sent to Novogene for library construction and sequencing. Bacterial and archaeal 16S rRNA genes (V4–V5 region) were amplified from genomic DNA using primers 515f and 806r with unique barcodes. Sequencing was performed on the Illumina Novaseq 6000 platform (Novogene, Beijing, China), generating 250-bp paired-end reads at an average depth of 0.1 million reads per sample.

Data processing was conducted through EasyAmplicon (1) and standalone tools. Paired reads were merged using fastq_mergepairs, with adapter and barcode trimming using fastq_stripleft and fastq_stripright. Quality filtering removed low-quality reads and singletons using fastq_maxee_rate and minuniquesize in VSEARCH 2.14.1 (2), and chimeric sequences were eliminated via uchime_ref. Amplicon sequence variants (ASVs) were generated with usearch_global, and taxonomic classification was assigned using the RDP 16S V18 database. Non-bacterial sequences were excluded based on taxonomy.

Alpha diversity indices (coverage, Chao1 richness, Shannon index, and inverse Simpson index) were calculated with the vegan R package (version 2.6.4), and rarefaction curves visualized sequencing depth completeness using the amplicon R package (version 1.14.2). Principal Coordinate Analysis (PCoA) was conducted on an unweighted UniFrac distance matrix with USEARCH (3) to assess beta diversity. Genus-level taxonomic composition was visualized using tax_stackplot, and paired samples were displayed in stacked bar charts. Statistical analysis was performed using STAMP 2.1.3 (4) with Welch's t-test for group comparisons (significance at *p* < 0.05), and extended error bar plots were generated to highlight significant differences. Unclassified reads were excluded from analyses at each taxonomic level.

**Pathogenicity Assay**

Pathogenicity assays were performed following previously established methods (5–7) on fully expanded immature leaves of grapefruit, prepared and inoculated within a quarantine greenhouse facility. Post-inoculation, plants were kept in an isolated greenhouse, and images were captured at specified intervals. To assess bacterial populations in the plants, three leaf discs (0.6 cm diameter) were collected using a leaf punch and homogenized in 1 ml of sterile water. Serial dilutions of these suspensions were plated on NA media for colony-forming unit (CFU) counts.

For the control efficacy of propionate against citrus canker, approximately 10-week-old immature grapefruit plants were used. The leaves were sprayed with a bacterial suspension (10^8^ CFU ml^-1^) of wild-type Xcc CQ13 on both sides before applying propionate around its EC50 concentration. For the preventive assay, leaves were first sprayed with propionate around its EC50 concentration, followed by the bacterial suspension (10^8^ CFU ml^-1^) of Xcc CQ13. The commercial Bordeaux mixture (80%) was diluted for 1:500 and sprayed in a similar way as a control agent. Petioles were wrapped in moist cotton and placed in a covered cultivation dish to maintain high humidity levels. In both assays, sterile water was used as a negative control, while wild-type Xcc CQ13 (10^8^ CFU ml^-1^) served as a positive control. Inoculated leaves were kept in a greenhouse at approximately 28–32 °C with over 80% relative humidity. Disease incidence was recorded across three independent bioassays, and the preventive efficacy of propionate was quantified by counting lesion numbers.

**RNA extraction, qRT-PCR and RNA-seq Analysis**

The Xcc CQ13 strain was cultured overnight in NB medium at 28°C with shaking at 200 rpm. The optical density at 600 nm (OD600) of the bacterial culture reached 0.5 in a 3 ml volume. The bacterial cultures were then treated with 0.5 mM and 50 mM sodium propionate for 1 hour. Untreated sample was served as control. RNA was extracted using the RNAprep Pure Cell/Bacteria Kit (TIANGEN) following the addition of RNAprotect Bacteria Reagent (Qiagen, Valencia, CA, U.S.A.) to stabilize the samples. For qRT-PCR analysis, genomic DNA was removed, and the RNA was reverse-transcribed into cDNA using the HIScript III 1st Strand cDNA Synthesis Kit (Vazyme). qRT-PCR assays were performed on a real-time PCR system using the ChamQ Universal SYBR qPCR Master Mix (Vazyme). The gyrB gene served as the endogenous control. Relative gene expression levels were calculated using the 2^−ΔΔCt^ method. For RNA-seq analysis, total RNA samples were sent to MagiGene (Guangzhou, China) for sequencing on the Illumina NovaSeq PE150 platform. The raw sequencing data were trimmed using fastp v0.23.2, and index establishment and sequence mapping were performed with HISAT2 v2.2.1 and SAMtools v1.16.1. Differentially expressed genes (DEGs) were identified using DEGSeq with the criteria of |Fold Change| > 1 and FDR < 0.05 (8). Principal Component Analysis (PCA), and Gene Set Enrichment Analysis (GSEA) were conducted using Omicshare, an interactive online data analysis platform, with significance set at p-value < 0.05.

**Bacterial Motility Assay**

Bacterial motility was assessed on semisolid nutrient agar (NA) plates containing 0.28% agar and supplemented with either 0 mM, 30 mM, or 100 mM sodium propionate. Bacteria were initially cultured in nutrient broth (NB) overnight at 200 rpm. Cultures were then centrifuged, washed, and diluted in sterile water to an OD600 of 0.5. A 5 µL aliquot of each bacterial suspension was spotted at the center of the plates, which were incubated at 28 °C. After 72 hours, plates were photographed to document motility. The assay was conducted three times independently, with each experiment performed in triplicate, yielding consistent results across replicates.

**Membrane Potential (ΔΨ) Detection Assay**

The membrane potential (ΔΨ) of bacterial cells was evaluated using the BacLight Bacterial Membrane Potential Kit (Invitrogen) in combination with flow cytometry. Bacterial cells were diluted to approximately 1 x 10^6^ CFU ml^-1^ in phosphate-buffered saline (PBS, pH 7.4) with 10 mM sodium phosphate and 145 mM sodium chloride. The PBS solution was sterilized by filtration through a 0.22 µm membrane filter. For the depolarized control, 10 μl of 500 μM carbonyl cyanide m-chlorophenylhydrazone (CCCP) in DMSO was added per ml of cell suspension and incubated for 15 minutes. For propionate stress conditions, cell samples were incubated for 15 minutes with one of the following: 100 μl of 500 mM sodium propionate, 100 μl of 200 mM potassium propionate, or 100 μl of 50 mM ammonium propionate. Following treatment, 1 ml of each cell suspension was stained with 10 µL of 3 mM DiOC_2_ (3) and incubated for 30 minutes at 30 °C. Stained cells were analyzed using a FACSCaliber flow cytometer (BD), with excitation at 488 nm and detection through 525-530 nm and 610 nm band-pass filters (approximately 20 nm bandwidth). Fluorescence signals were collected in both green and red channels, along with forward and side scatter, using logarithmic amplification. A ratiometric method, as outlined by Novo et al. (9), was employed to obtain cell-size-independent values for membrane potential. Each experiment included three independent biological replicates to ensure reliability.

**Intracellular pH experiment**

Intracellular pH was measured using the ratiometric fluorescent dye 2',7'-bis-(2-carboxyethyl)-5-(and-6)-carboxyfluorescein, acetoxymethyl ester (BCECF-AM; Beyotime), which fluoresces upon interaction with the cytoplasm. The ratio of fluorescence emissions at wavelengths of 488 nm and 440 nm was used to determine intracellular pH. To generate a standard curve for intracellular pH calibration, a series of solutions with fixed pH values ranging from 4 to 10 were prepared in NB medium, adjusting pH with HCl or NaOH as needed, and verified using a pH meter. Cultures of Xcc CQ13 were grown overnight under standard conditions and then diluted to an OD600 of 1. BCECF-AM was added to a final concentration of 2 µM, along with 50 µM carbonyl cyanide m-chlorophenylhydrazone (CCCP) to depolarize the membrane. This depolarization allowed the extracellular and intracellular pH to equilibrate, making the conditions suitable for standard curve generation. Over a 16-hour period, fluorescence equilibrium of BCECF-AM was ensured, and emission ratios at λem = 488 nm to λem = 440 nm (under excitation at λex = 535 nm) were measured. These ratios provided data points for constructing the standard curve. Subsequently, ratiometric measurements of BCECF-AM under various propionate concentrations were taken, and intracellular pH was calculated by interpolating these ratios against the standard curve.

**Generation of mutant strains and complemented strains**

Deletion mutants were generated using a double-crossover homologous recombination technique, allowing for targeted gene deletion. The knockout strategy and validation results are shown in Fig. S8. Briefly, the genomic DNA template was extracted using a Genomic DNA Extraction Kit (TIANGEN). For each target gene (*prpR, prpB, prpC,* and *acnD*), the upstream and downstream flanking regions were amplified (Table S2). A Gibson reaction was performed to insert two fragments into the multiple cloning sites of the pNPTS138 suicide vector. The vector was transformed into Xcc CQ13 by electroporation and markerless deletion mutants were produced using a two-step sucrose counterselection procedure (10). To construct the complemented strains, DNA fragments covering the entire coding region and the promoter region of target genes were amplified and inserted into the multiple cloning site of the plasmid pL-2. Plasmids were introduced into corresponding mutant strains by electroporation.

**PrpR protein purification**

The full-length open reading frame (ORF) of *prpR* was cloned into the plasmid pMAL-c5x, resulting in the construct pMAL-c5x-prpR. This recombinant plasmid was then transformed into BL21 (DE3) competent cells using heat shock. A 500 ml culture of *E. coli* was grown in LB medium at 37°C to an optical density (OD600) of 0.5, followed by induction with 0.5 mM isopropyl-β-D-thiogalactopyranoside (IPTG) for 3 hours. After induction, the cells were harvested by centrifugation. The cell pellets were resuspended in purification buffer (50 mM Tris-HCl [pH 8.0], 200 mM NaCl, 100 mM KCl, 1 mM EDTA, 1 mM DTT) and disrupted using an ultrasonicator. To purify the MBP-PrpR protein, the cell lysate was incubated with amylose resin for 20 minutes. The amylose column was washed four to six times with purification buffer, using a volume five times that of the column. Proteins were eluted in purification buffer containing 10 mM maltose and 20% glycerol. The MBP protein, used as a control, was purified following the same procedure.

**Immunoblot**

The pL1-*PprpB*-mCherry-Flag plasmid was constructed and introduced into the wild-type Xcc CQ13 and the *prpR* mutant strain through electroporation. Cells were cultured in nutrient broth (NB) medium supplemented with spectinomycin at 28°C until an optical density (OD600) of 0.5 was reached. Sodium propionate was then either added to the cell culture or omitted, and the cultures were induced for 1 hour. Samples were collected every 30 minutes, continuing up to a total of 120 minutes. For each time point, 1 ml of bacterial culture was centrifuged, and the resulting pellet was resuspended in an appropriate volume of SDS-PAGE protein loading buffer (Beyotime). The samples were boiled at 100°C for 10 minutes to lyse the cells. Denatured protein samples were separated using 1× SDS Tris-Glycine buffer at 120 V with the TGX FastCast Acrylamide Kit (12% gel, Bio-Rad) for 45 minutes. Proteins were transferred onto PVDF membranes using a membrane transfer apparatus. Protein expression was detected using anti-FLAG antibody.

**Gel Shift Assays**

Interactions of recombinant PrpR protein with DNA fragments containing the promoter regions of prpB, prpC, acnD, and prpF were investigated using a gel shift assay. The respective DNA fragments were amplified by PCR with specific primers (see Table S2) and purified from agarose gels using the Microcolumn Concentrated DNA Gel Kit (ZOMANBIO) or obtained through primer cross-linking. For the gel shift assay, DNA probes (100-150 ng) were incubated at 25°C for 30 minutes with 2 µM purified PrpR protein, appropriately diluted in 1× Binding Buffer (50 mM Tris, pH 8.8; 50 mM NaCl; 10 mM MgCl₂; 0.5 mM EDTA; 0.5 mM DTT; 10% glycerol). The resulting nucleoprotein complexes were resolved on 12% polyacrylamide gels in 1× Tris-glycine buffer at 25 V. The gels were stained with ethidium bromide (EB) solution and visualized using a gel imaging system (Biotree).

**SCFAs targeting metabolome analysis**

Soil samples were collected from a citrus grove under orange trees in Heyuan City, Guangdong Province, China. The soil was divided into six portions, each weighing 500 g. Three of these portions underwent high-temperature and high-pressure sterilization. Subsequently, 30 mL of 50 mM sodium propionate was added to each portion of soil, and the samples were placed in a 30°C incubator. Sampling was conducted at 0 and 30 days. For LC-MS/MS analysis, 50 mg of each soil sample was weighed and diluted to an appropriate volume with 80% methanol. The mixture was vortexed and centrifuged, and 20 μL of the supernatant was transferred to a 1.5 mL centrifuge tube. N-(3-dimethylaminopropyl)-N'-ethylcarbodiimide hydrochloride (EDC) solution and 3-nitrophenylhydrazine (3-NPH) were added for derivatization. The initial mobile phase solution was added to bring the total volume to 500 μL, followed by vortex mixing. Then, 200 μL of the resulting solution was transferred to a sample vial for LC-MS/MS detection. Detection was performed using an AB Sciex 4500MD triple quadrupole mass spectrometer coupled with the Jasper liquid chromatography system. The chromatography column used was an Agilent Poroshell 120 EC-C18 (2.7 μm, 2.1 × 100 mm) at a column temperature of 40°C, with an injection volume of 2 μL. The mobile phase consisted of A: Water and B: Methanol + Acetonitrile (1:1). Mass spectrometry was conducted in multiple reaction monitoring (MRM) mode with negative ion detection. Quantitative analysis was performed using an internal standard method, with a standard chromatogram of seven fatty acids as the reference. Internal standard metabolites, including acetic acid, isobutyric acid, and butyric acid, were purchased from CATO, while propionic acid was obtained from AccuStandard. Valeric acid, isovaleric acid, and hexanoic acid were purchased from BePure.

**References:**

1. Liu Y-X, Chen L, Ma T, Li X, Zheng M, Zhou X, Chen L, Qian X, Xi J, Lu H, Cao H, Ma X, Bian B, Zhang P, Wu J, Gan R-Y, Jia B, Sun L, Ju Z, Gao Y, Wen T, Chen T. 2023. EasyAmplicon: An easy-to-use, open-source, reproducible, and community-based pipeline for amplicon data analysis in microbiome research. iMeta 2:e83.

2. Rognes T, Flouri T, Nichols B, Quince C, Mahé F. 2016. VSEARCH: a versatile open source tool for metagenomics. PeerJ 4:e2584.

3. Edgar RC. 2010. Search and clustering orders of magnitude faster than BLAST. Bioinforma Oxf Engl 26:2460–2461.

4. Parks DH, Tyson GW, Hugenholtz P, Beiko RG. 2014. STAMP: statistical analysis of taxonomic and functional profiles. Bioinforma Oxf Engl 30:3123–3124.

5. Guo Y, Figueiredo F, Jones J, Wang N. 2011. HrpG and HrpX Play Global Roles in Coordinating Different Virulence Traits of Xanthomonas axonopodis pv. citri. Mol Plant-Microbe Interactions® 24:649–661.

6. Li J, Wang N. 2011. The wxacO gene of Xanthomonas citri ssp. citri encodes a protein with a role in lipopolysaccharide biosynthesis, biofilm formation, stress tolerance and virulence. Mol Plant Pathol 12:381–396.

7. Yan Q, Wang N. 2012. High-throughput screening and analysis of genes of Xanthomonas citri subsp. citri involved in citrus canker symptom development. Mol Plant-Microbe Interact MPMI 25:69–84.

8. Wang L, Feng Z, Wang X, Wang X, Zhang X. 2010. DEGseq: an R package for identifying differentially expressed genes from RNA-seq data. Bioinforma Oxf Engl 26:136–138.

9. Novo D, Perlmutter NG, Hunt RH, Shapiro HM. 1999. Accurate flow cytometric membrane potential measurement in bacteria using diethyloxacarbocyanine and a ratiometric technique. Cytometry 35:55–63.

10. Zhou X, Hu X, Li J, Wang N. 2015. A Novel Periplasmic Protein, VrpA, Contributes to Efficient Protein Secretion by the Type III Secretion System in Xanthomonas spp. Mol Plant-Microbe Interact MPMI 28:143–153.
