# Supplementary material for: Propionate serves as a degradable control agent of citrus canker by acidifying cytoplasm and depleting intracellular ATP in Xanthomonas citri
Source: mBio. 2025 Apr 29;16(6):e00642-25. doi: 10.1128/mbio.00642-25 (PMC12153269; doi:10.1128/mbio.00642-25)

Fig. S1

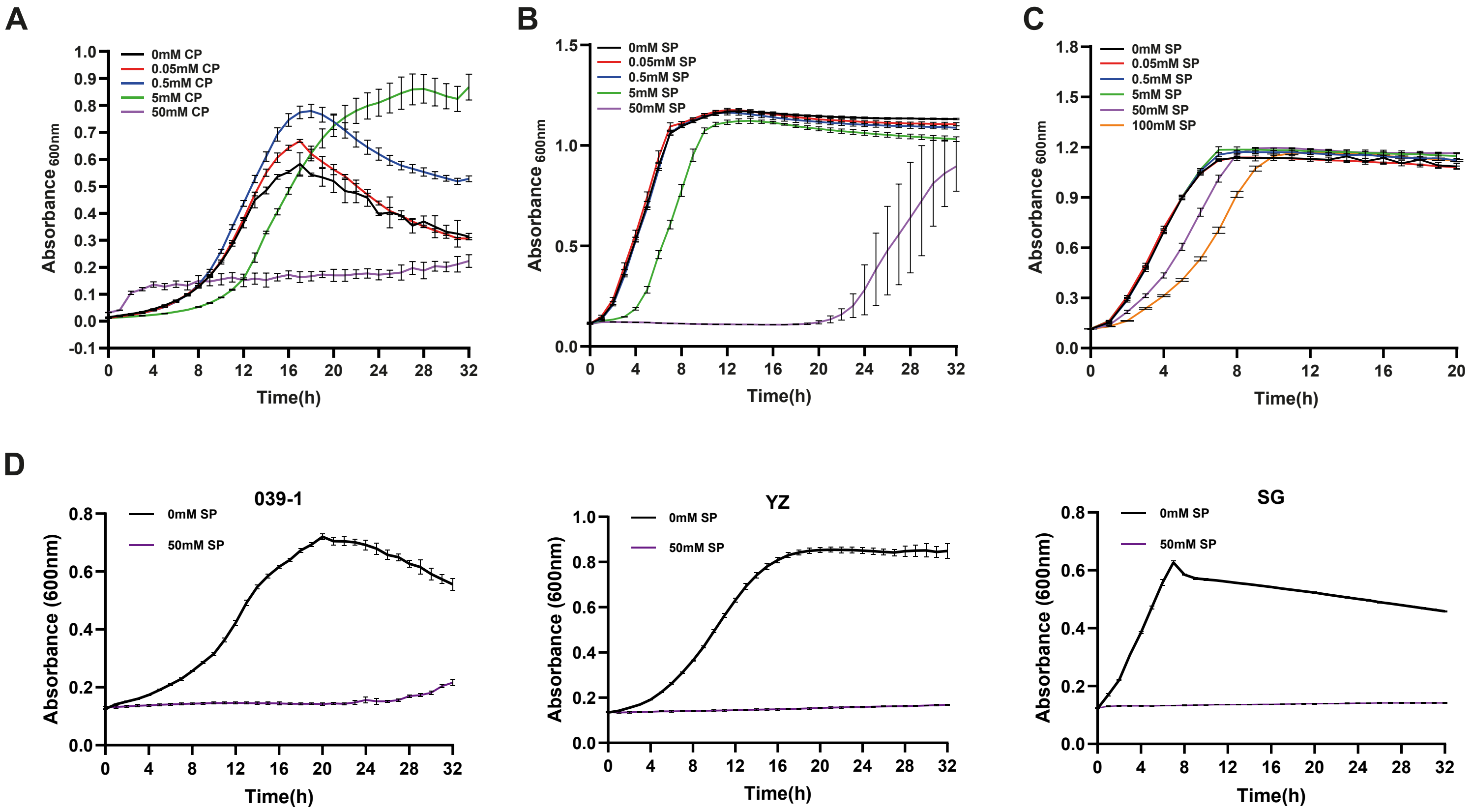
A
0mM CP
0.05mM CP
0.5mM CP
5mM CP
50mM CP
Absorbance 600nm
Time(h)
B
0mM SP
0.05mM SP
0.5mM SP
5mM SP
50mM SP
Absorbance 600nm
Time(h)
C
0mM SP
0.05mM SP
0.5mM SP
5mM SP
50mM SP
100mM SP
Absorbance 600nm
Time(h)
D
039-1
0mM SP
50mM SP
Absorbance (600nm)
Time(h)
YZ
0mM SP
50mM SP
Absorbance (600nm)
Time(h)
SG
0mM SP
50mM SP
Absorbance (600nm)
Time(h)

Fig. S2

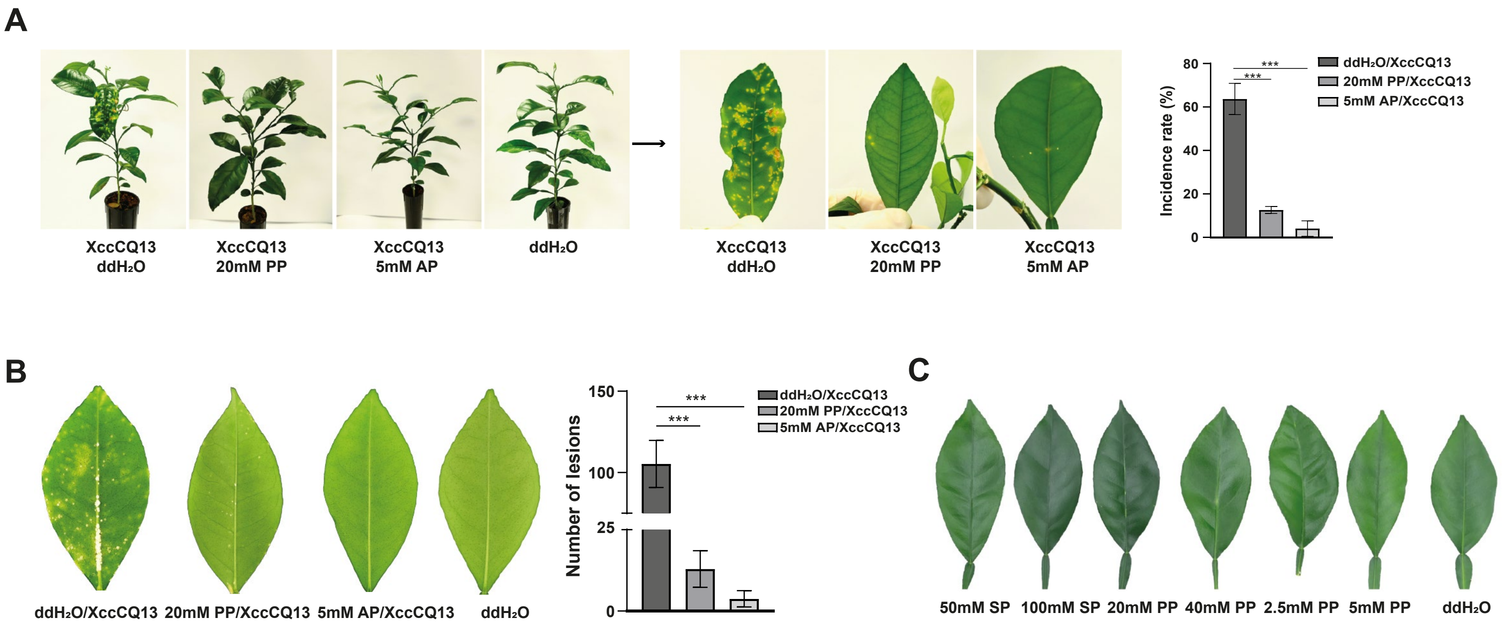
A
XccCQ13
ddH₂O
XccCQ13
20mM PP
XccCQ13
5mM AP
ddH₂O
XccCQ13
ddH₂O
XccCQ13
20mM PP
XccCQ13
5mM AP
Incidence rate (%)
0
20
40
60
80
***
***
ddH₂O/XccCQ13
20mM PP/XccCQ13
5mM AP/XccCQ13
B
ddH₂O/XccCQ13
20mM PP/XccCQ13
5mM AP/XccCQ13
ddH₂O
Number of lesions
0
25
100
150
***
***
ddH₂O/XccCQ13
20mM PP/XccCQ13
5mM AP/XccCQ13
C
50mM SP
100mM SP
20mM PP
40mM PP
2.5mM PP
5mM PP
ddH₂O

Fig. S3

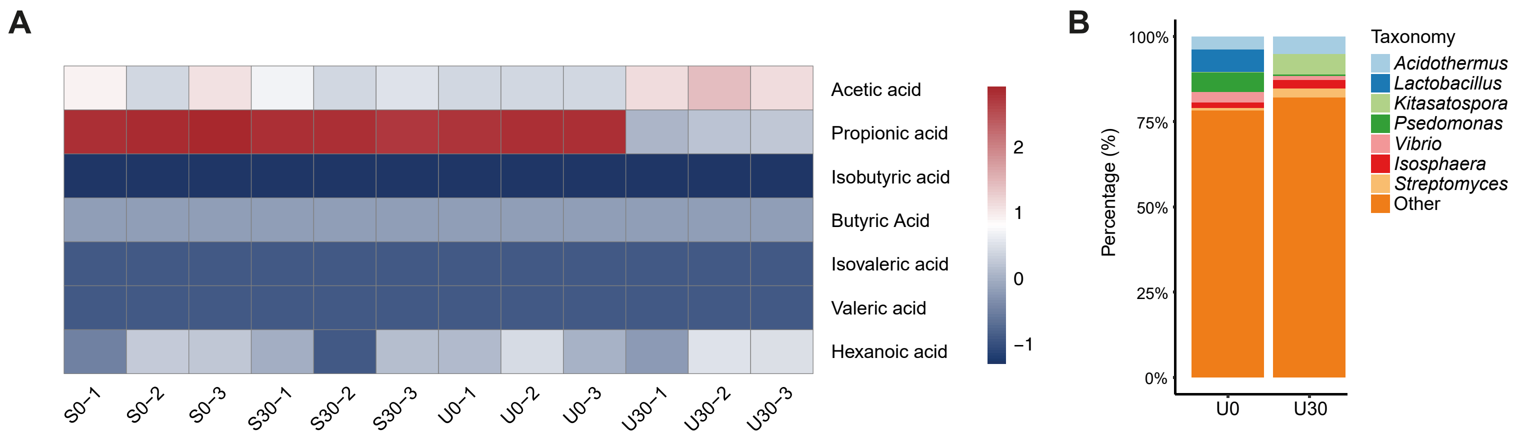
A
Acetic acid
Propionic acid
Isobutyric acid
Butyric Acid
Isovaleric acid
Valeric acid
Hexanoic acid
2
1
0
−1
S0−1
S0−2
S0−3
S30−1
S30−2
S30−3
U0−1
U0−2
U0−3
U30−1
U30−2
U30−3
B
100%
75%
50%
25%
0%
Percentage (%)
U0
U30
Taxonomy
Acidothermus
Lactobacillus
Kitasatospora
Psedomonas
Vibrio
Isosphaera
Streptomyces
Other

Fig. S4

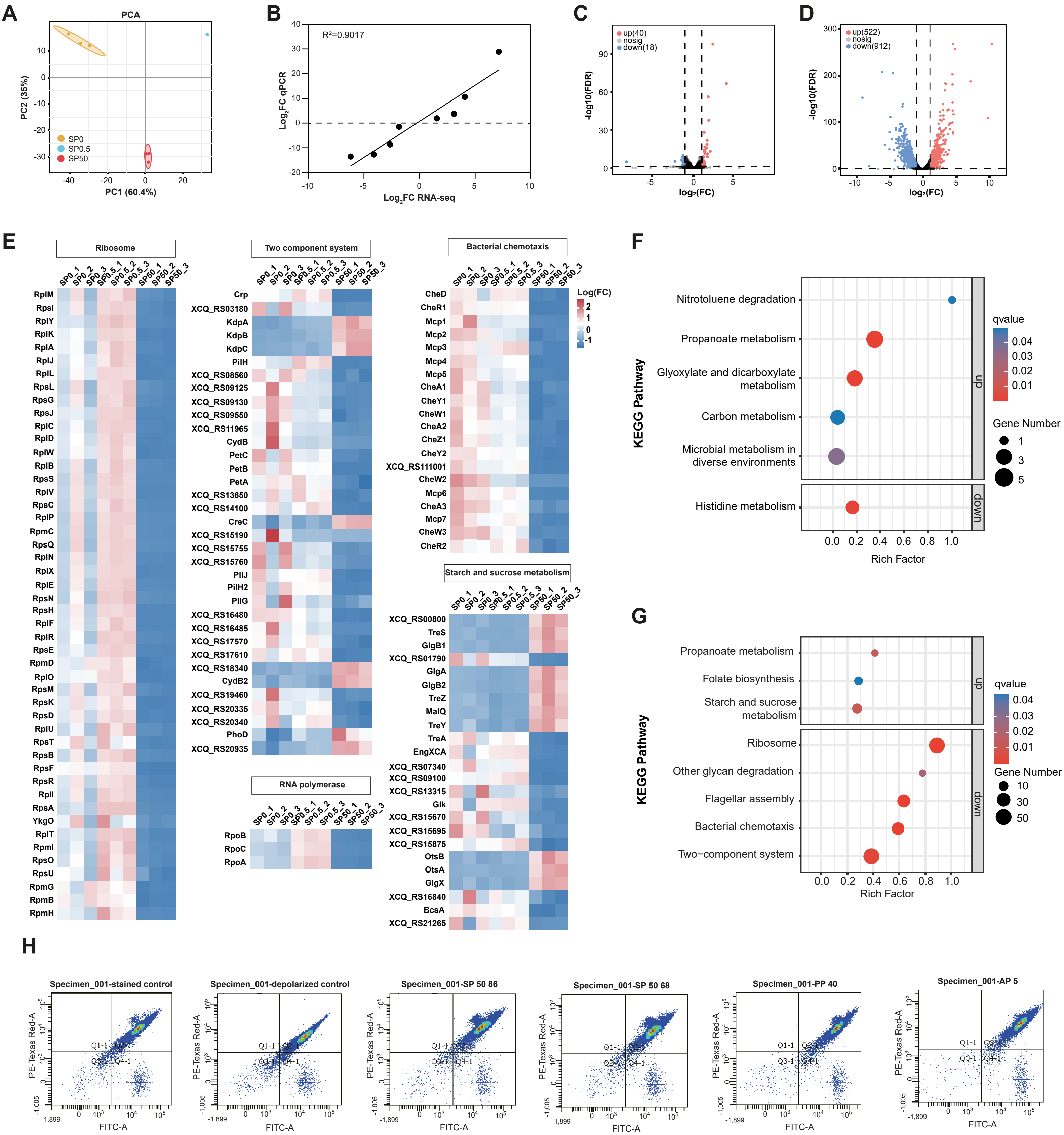

A
PCA
PC1 (60.4%)
PC2 (35%)
SP0
SP0.5
SP50
B
R²=0.9017
Log2FC qPCR
Log2FC RNA-seq
C
up(40)
nosig
down(18)
-log10(FDR)
log2(FC)
D
up(522)
nosig
down(912)
-log10(FDR)
log2(FC)
E
Ribosome
Two component system
Bacterial chemotaxis
Starch and sucrose metabolism
RNA polymerase
Log(FC)
F
KEGG Pathway
Nitrotoluene degradation
Propanoate metabolism
Glyoxylate and dicarboxylate metabolism
Carbon metabolism
Microbial metabolism in diverse environments
Histidine metabolism
up
down
qvalue
Gene Number
Rich Factor
G
KEGG Pathway
Propanoate metabolism
Folate biosynthesis
Starch and sucrose metabolism
Ribosome
Other glycan degradation
Flagellar assembly
Bacterial chemotaxis
Two−component system
up
down
qvalue
Gene Number
Rich Factor
H
Specimen_001-stained control
Specimen_001-depolarized control
Specimen_001-SP 50 86
Specimen_001-SP 50 68
Specimen_001-PP 40
Specimen_001-AP 5
PE-Texas Red-A
FITC-A
Q1-1
Q2-1
Q3-1
Q4-1

Fig. S5

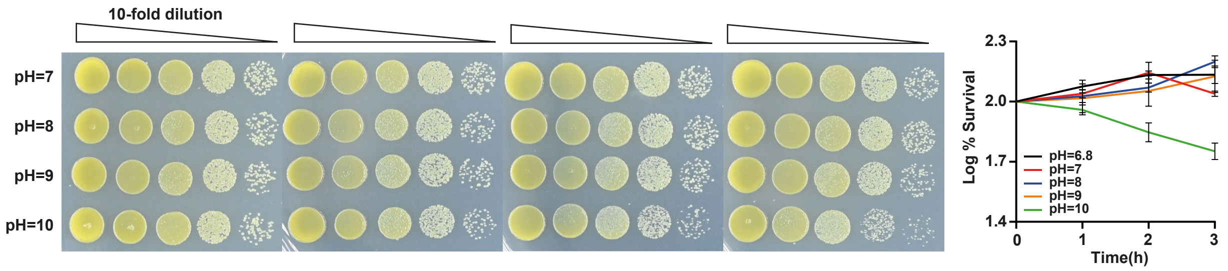
10-fold dilution
pH=7
pH=8
pH=9
pH=10
Log % Survival
2.3
2.0
1.7
1.4
0
1
2
3
Time(h)
pH=6.8
pH=7
pH=8
pH=9
pH=10

Fig. S6

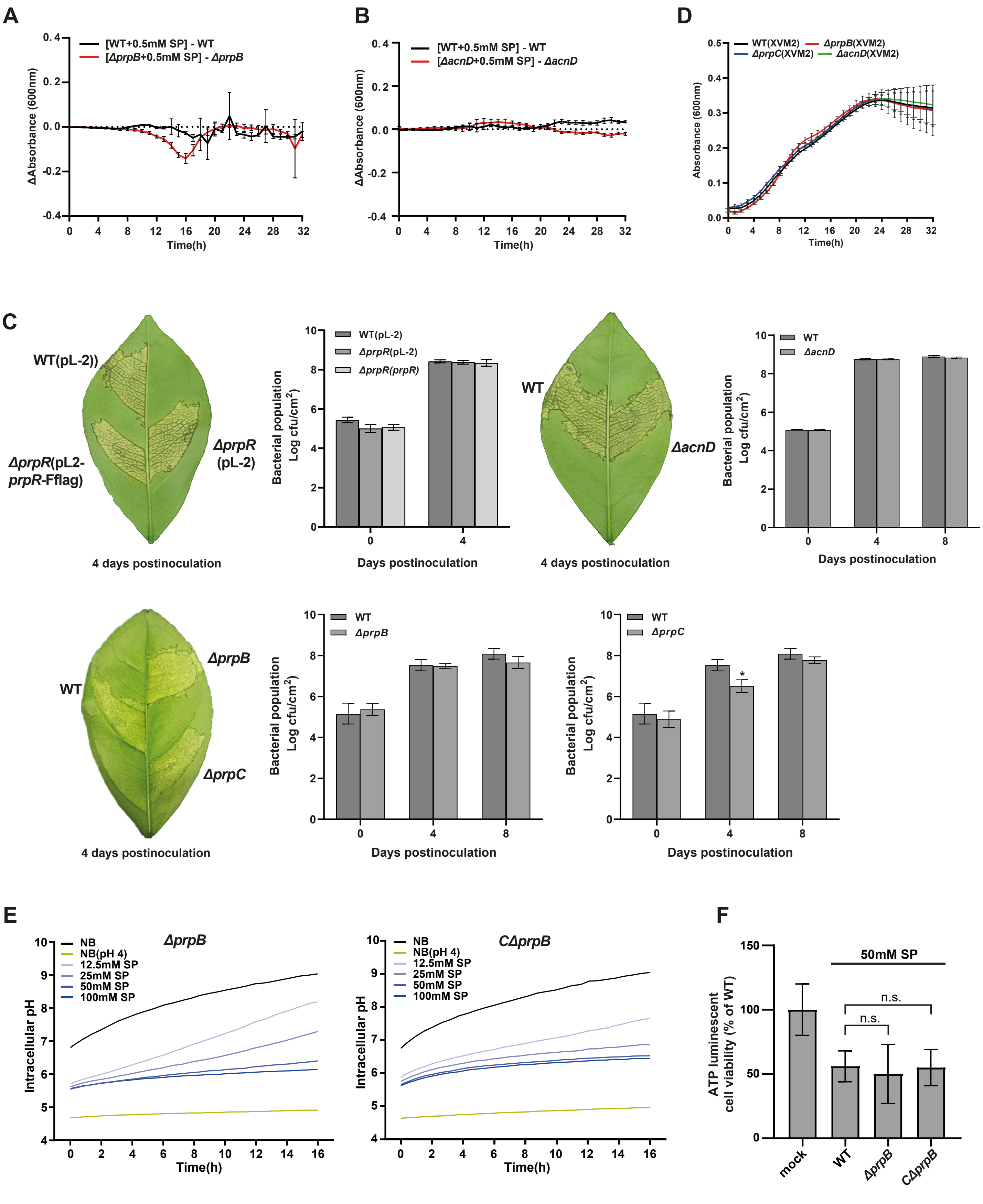
A
[WT+0.5mM SP] - WT
[ΔprpB+0.5mM SP] - ΔprpB
ΔAbsorbance (600nm)
Time(h)
B
[WT+0.5mM SP] - WT
[ΔacnD+0.5mM SP] - ΔacnD
ΔAbsorbance (600nm)
Time(h)
D
WT(XVM2)
ΔprpB(XVM2)
ΔprpC(XVM2)
ΔacnD(XVM2)
Absorbance (600nm)
Time(h)
C
WT(pL-2))
ΔprpR(pL2-prpR-Fflag)
ΔprpR (pL-2)
4 days postinoculation
WT(pL-2)
ΔprpR(pL-2)
ΔprpR(prpR)
Bacterial population Log cfu/cm2)
Days postinoculation
WT
ΔacnD
4 days postinoculation
WT
ΔacnD
Bacterial population Log cfu/cm2)
Days postinoculation
ΔprpB
WT
ΔprpC
4 days postinoculation
WT
ΔprpB
Bacterial population Log cfu/cm2)
Days postinoculation
WT
ΔprpC
Bacterial population Log cfu/cm2)
Days postinoculation
E
ΔprpB
NB
NB(pH 4)
12.5mM SP
25mM SP
50mM SP
100mM SP
Intracellular pH
Time(h)
CΔprpB
NB
NB(pH 4)
12.5mM SP
25mM SP
50mM SP
100mM SP
Intracellular pH
Time(h)
F
50mM SP
n.s.
n.s.
ATP luminescent cell viability (% of WT)
mock
WT
ΔprpB
CΔprpB

Fig. S7

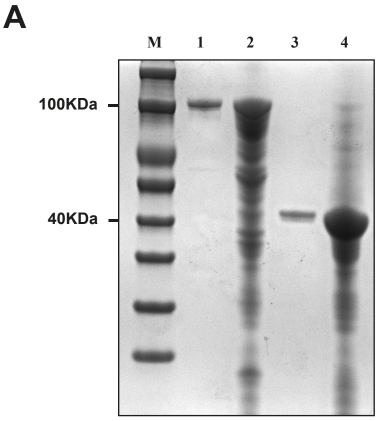
A
M
1
2
3
4
100KDa
40KDa

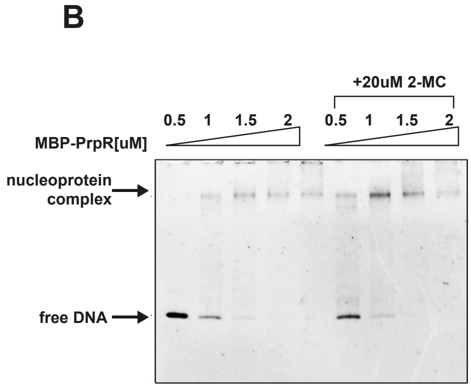
B
+20uM 2-MC
0.5
1
1.5
2
0.5
1
1.5
2
MBP-PrpR[uM]
nucleoprotein complex
free DNA

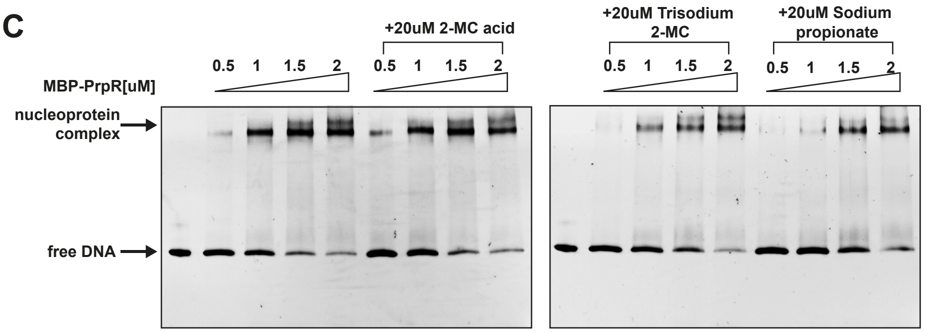
C
+20uM 2-MC acid
+20uM Trisodium 2-MC
+20uM Sodium propionate
0.5
1
1.5
2
MBP-PrpR[uM]
nucleoprotein complex
free DNA

Fig. S8

A

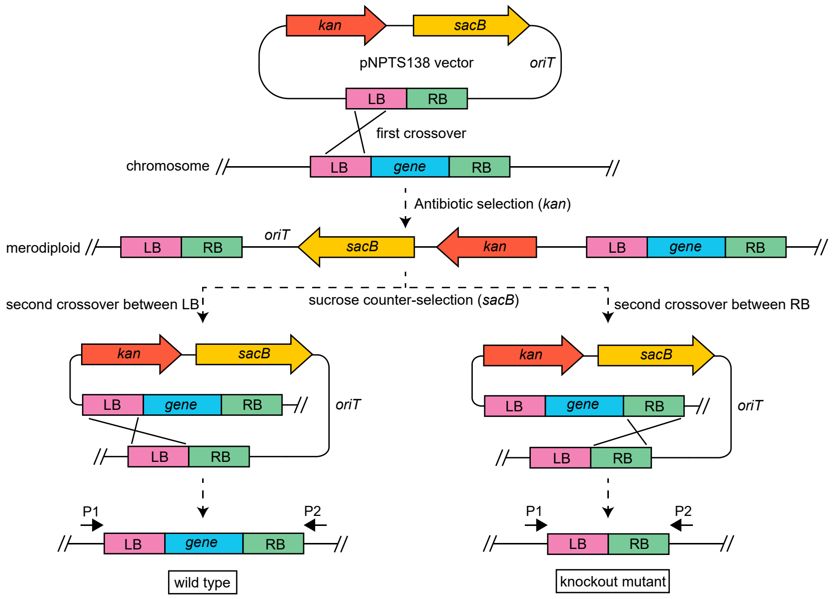

Target gene	Length of target gene	Length of LB	Length of RB	Length PCR product (P1+P2) using WT genomic DNA	Length PCR product (P1+P2) using mutant genomic DNA
prpR	1617	905	958	3996	2379
prpB	897	908	845	3624	2727
prpC	1155	902	871	3350	2195
acnD	2592	1360	1343	4847	2255

(Unit: bp)

B

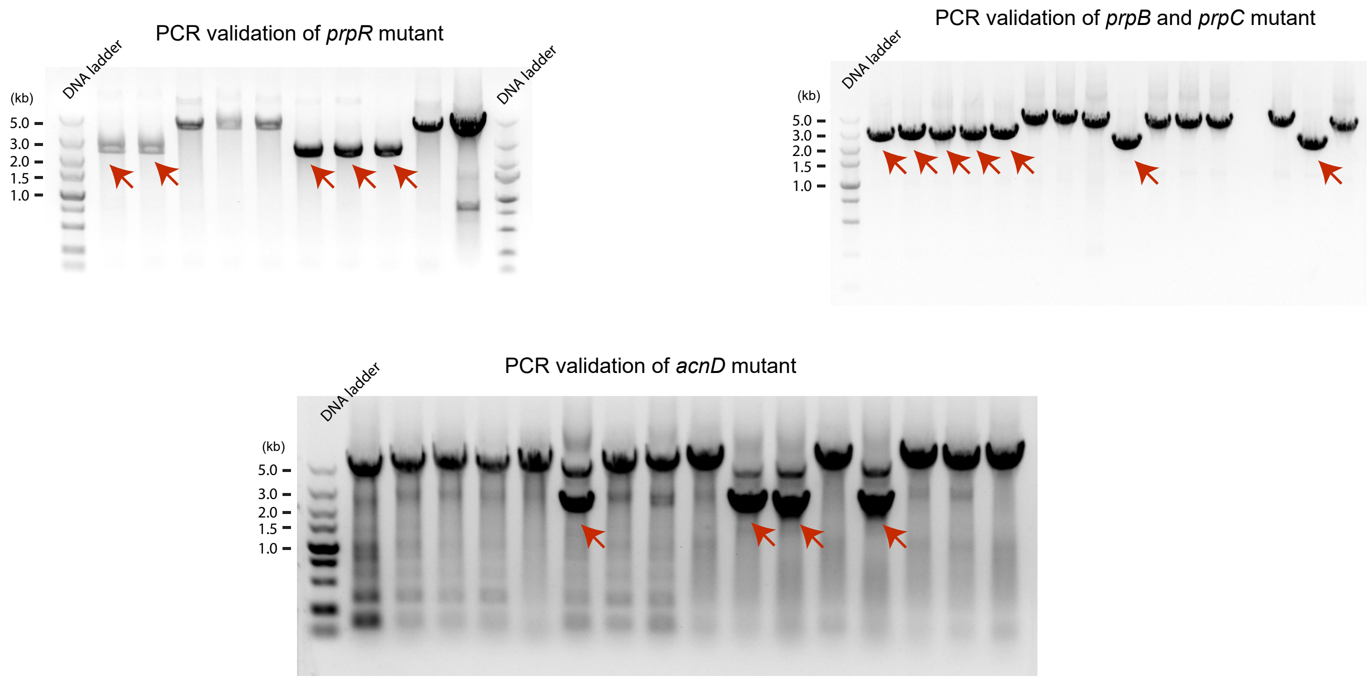

Supplement: Supplemental figures — Fig. S1 to S8. [file mbio.00642-25-s0002.pdf]
