# Supplementary material for: Propionate serves as a degradable control agent of citrus canker by acidifying cytoplasm and depleting intracellular ATP in Xanthomonas citri
Source: mBio. 2025 Apr 29;16(6):e00642-25. doi: 10.1128/mbio.00642-25 (PMC12153269; doi:10.1128/mbio.00642-25)
Supplement: Legends — Supplemental figure and movie legends. [file mbio.00642-25-s0003.docx]

**Supplementary figure legend**

**Figure S1. The inhibition of propionate to different bacteria.**

(A) Growth curve of Xcc CQ13 in nutrient broth (NB) supplemented with a ten-fold concentration gradient of calcium propionate, ranging from 0.05 mM to 50 mM. Cultures were grown overnight and diluted to an OD600 of 0.03 in NB medium containing calcium propionate. (B) Growth curve of *P. brasiliense* SX309 in NB supplemented with a ten-fold concentration gradient of sodium propionate, from 0.05 mM to 50 mM. Cells were grown overnight and diluted to an OD600 of 0.03 in NB medium with sodium propionate. (C) Growth curve of *E. coli* DH5α in Luria-Bertani (LB) medium supplemented with a ten-fold concentration gradient of sodium propionate, from 0.05 mM to 100 mM. Cultures were grown overnight and diluted to an OD600 of 0.03 in LB medium containing sodium propionate. All experiments were conducted in 24-well plates using a Tecan microplate reader. Data represent the mean and standard deviation (SD) from at least two independent experiments, each performed with three biological replicates (n=6). (D) Growth curves of Xcc 039-1, Xcc YZ and Xcc SG in nutrient broth (NB) medium supplemented with 50 mM SP. Data represent the mean and SD of at least two independent experiments with three biological replicates each (n = 6).

**Figure S2. PP and AP effectively provide both control and prevention for citrus canker disease management.**

(A) Xcc cells were applied to grapefruit plants at a concentration of 10^8^ CFU/mL, followed by PP (20 mM) or AP (2.5 mM) treatment at 0.5, 3-, and 6-days post-inoculation. Plants and leaves were photographed 25 days after Xcc inoculation. The left panel shows the overall plant condition, while the right panel presents close-ups of diseased leaves and statistical analysis of disease incidence rates. (B) PP (20 mM) or AP (2.5 mM) was applied to grapefruit plants before Xcc inoculation (10^8^ CFU/mL). Treated leaves were incubated in a petri dish with 90% humidity at 30°C for two weeks and photographed 15 days post-inoculation. (C) Different types of propionate (sodium, potassium, and ammonium) were applied to grapefruit leaves at concentrations equivalent to their respective EC50 or twice the EC50. Leaves were photographed 30 days after treatment. Each experiment was repeated at least three times with consistent results; one representative leaf is shown. Mean and SD are from two independent experiments, each with a minimum of six leaves. Three asterisks denote significant differences (*p* < 0.001) according to a t-test.

**Figure S3. Propionate as a degradable antimicrobial compound.**

(A) Heatmap of short-chain fatty acid (SCFA) levels from targeted metabolomics analysis. Three biological replicates per treatment group were analyzed. (B) Bar chart displaying relative abundance of microbial communities at the genus level in sodium propionate-treated (U30) and untreated (U0) soil samples.

**Figure S4. Sodium propionate inhibits protein synthesis, two-component system, bacterial chemotaxis, starch and sucrose metabolism, and RNA polymerase in Xcc CQ13.**

(A) Principal Component Analysis (PCA) of the overall gene expression profile for untreated wild-type Xcc CQ13 (SP0), Xcc treated with 0.5 mM sodium propionate (SP0.5), and Xcc treated with 50 mM sodium propionate (SP50). (B) Validation of RNA-seq expression patterns with qPCR analysis. Correlation between qPCR and RNA-seq results shows Pearson’s r = 0.9017 (*p* < 0.01). (C) Volcano plot of differentially expressed genes in Xcc CQ13 treated with 0.5 mM sodium propionate (SP0.5) vs. untreated (SP0). (D) Volcano plot of differentially expressed genes in Xcc CQ13 treated with 50 mM sodium propionate (SP50) vs. untreated (SP0). (E) Heatmap of gene expression profiles related to ribosome function, two-component system, bacterial chemotaxis, starch and sucrose metabolism, and RNA polymerase synthesis. (F) KEGG enrichment analysis of pathways for SP0.5 treatment (q <0.05), with circle color indicating q-value (red = low, blue = high) and circle size proportional to the number of enriched genes. (G) KEGG enrichment analysis for SP50 treatment (q < 0.05) with similar color and size indications as in (F). (H) Flow cytometric analysis of Xcc CQ13 cells treated with different propionates, shown in FITC-A and PE-Texas Red-A dot plots. Sequence of images (left to right) represents: stain control, membrane depolarization control, 50 mM SP at pH 8.6, 50 mM SP at pH 6.8, 40 mM PP at pH 6.3, and 5 mM AP at pH 4.9.

**Figure S5. Cell viability assay of Xcc CQ13 treated by alkaline culture medium.**

Xcc CQ13 cells were cultured overnight and diluted to an OD600 of 0.5. The original medium was replaced with fresh NB medium adjusted to pH levels ranging from 7 to 10. The cells were then incubated for 3 minutes, 1 hour, 2 hours, and 3 hours. Following incubation, a 10-fold gradient dilution was performed, and the bacterial suspension was spread on NA plates for colony-forming unit (CFU) counting. The original pH of NB medium, which is 6.8, served as the unadjusted control. Survival percentages were calculated by comparing treated cells to untreated cells. Data are presented as the Mean ± SD derived from at least two independent experiments, each with three biological replicates (n=6).

**Figure S6. Responses of *prp* genes to propionate and their roles in virulence.**

(A and B) The sensitivity of *prpB* and *acnD* mutants to 0.5 mM sodium propionate. ΔAbsorbance represents the difference in absorbance values between strains cultured in NB medium supplemented with 0.5 mM sodium propionate and those cultured in standard NB medium. (C) Pathogenicity assay for *prp* mutants in citrus host. Cells were inoculated onto grapefruit leaves by injection at 10^8^ CFU ml^-1^. Leaves were photographed 4 days post-inoculation. Pathogenicity assays were repeated three times with similar results, and only one representative leaf was represented. For bacterial population, cells were recovered from the inoculated leaves at 0, 4, 8 days post-inoculation, and the values are the means from three repeats. Mean and SD are plotted. An asterisk indicates a significant difference (*p* < 0.01) by t-test. (D) Growth curves of wild type, *ΔprpB*, *ΔprpC*, *ΔacnD* in XVM2 medium. Cultures were grown overnight, diluted to an OD600 of 0.03, and incubated in XVM2 medium. Experiments were conducted in 24-well plates using a Tecan microplate reader. Data represent the mean and SD of at least two independent experiments with three biological replicates each (n = 6). (E) Intracellular pH of *ΔprpB* and complementation strain. Cells were cultured to OD600 = 1 and transferred to fresh NB medium with 50 mM SP. BCECF-AM (2 μM) and CCCP (50 μM) were added for fluorescence ratiometric pH measurements at λem = 488 nm and λem = 440 nm under λex = 535 nm. A ratio-pH standard curve was generated for absolute intracellular pH determination. Propionate treatment was initiated for 15 min, followed by BCECF-AM addition, and fluorescence intensity was monitored for 16 hours. Data represent three independent biological duplicates. (F) Intracellular ATP assay. Strains were treated with 50 mM SP. ATP luminescence was measured to assess viability (Mean ± SD, n = 5). Significance is denoted by two asterisks (p < 0.001) using a t-test.

**Figure S7. Purification of PrpR protein and its binding to potential ligands.**

(A) SDS-PAGE gel showing protein samples at various stages of purification. Lanes include: M, molecular mass markers; 1, purified MBP-PrpR protein obtained through affinity column chromatography; 2, crude protein extract from *E. coli* (pMAL-c5x-prpR); 3, purified MBP protein obtained through affinity column chromatography; 4, crude protein extract from *E. coli* (pMAL-c5x). (B and C) Analysis of ligand binding for PrpR transcription factors via gel shift assays.

**Figure S8. Gene knockout strategy and PCR validation of *prpR*, *prpB*, *prpC*, and *acnD* mutants.**

(A) Schematic representation of the gene knockout strategy using a double-crossover homologous recombination approach. The table summarizes the lengths of the left and right homologous arms (LB and RB) and the expected PCR validation fragment sizes for *prpR*, *prpB*, *prpC*, and *acnD*. (B) Agarose gel electrophoresis results confirming gene deletions through PCR validation of *prpR*, *prpB*, *prpC*, and *acnD* mutant strains.

**Movie S1. Times-lapse microscopy of Xcc CQ13 wild type strain grown in the XVM2 agarose pad. Images were taken every 20 minutes. This movie is corresponding to Fig. 1G.**

**Movie S2. Times-lapse microscopy of Xcc CQ13 wild type strain grown in the XVM2 agarose pad supplemented with 50 mM SP. Images were taken every 20 minutes. This movie is corresponding to Fig. 1G.**

**Movie S3. Times-lapse microscopy of Xcc CQ13 wild type strain grown in the XVM2 agarose pad supplemented with 20 mM PP. Images were taken every 20 minutes. This movie is corresponding to Fig. 1G.**

**Movie S4. Times-lapse microscopy of Xcc CQ13 wild type strain grown in the XVM2 agarose pad supplemented with 5 mM AP. Images were taken every 20 minutes. This movie is corresponding to Fig. 1G.**
