# Supplementary material for: Propionate serves as a degradable control agent of citrus canker by acidifying cytoplasm and depleting intracellular ATP in Xanthomonas citri
Source: mBio. 2025 Apr 29;16(6):e00642-25. doi: 10.1128/mbio.00642-25 (PMC12153269; doi:10.1128/mbio.00642-25)
Supplement: Table S1 — Strains and plasmids. [file mbio.00642-25-s0004.docx]

**Table S1.** Strains and plasmids used in this study.

| **Types** | **Relevant characteristic** | **Reference or source** |
| --- | --- | --- |
| **strains** |  |  |
| *Escherichia coli* |  |  |
| DH5α |  | Shaprio shock |
| *Xanthomonas citri* subsp. *citri* |  |  |
| XccCQ13 | wild type; Rif^r^ | (Wu et al. 2022) |
| XccCQ13*ΔprpR* | *prpR* gene deletion strain in the background of XccCQ13; | This study |
| XccCQ13*ΔprpB* | *prpB* gene deletion strain in the background of XccCQ13; | This study |
| XccCQ13*ΔprpC* | *prpC* gene deletion strain in the background of XccCQ13; | This study |
| XccCQ13*ΔacnD* | *acnD* gene deletion strain in the background of XccCQ13; | This study |
| **plasmids** |  |  |
| pNPTS138 | sacB , Suicide vector. Kn^R^ |  |
| pBBR1MCS-2 | Broad host expression vector. Kn^R^ | (Kovach et al. 1995) |
| pUFR034 | Shuttle vector. Kn^R^ | (DeFeyter, Kado, and Gabriel 1990) |
| pMAL-c5x |  |  |
| pNPTS138-*prpR* | pNPTS138 derivative contacting the flanking regions of *prpR* (XCQ_05765). Kn^R^ | This study |
| pNPTS138-*prpB* | pNPTS138 derivative contacting the flanking regions of *prpB* (XCQ_05770). Kn^R^ | This study |
| pNPTS138-*prpC* | pNPTS138 derivative contacting the flanking regions of *prpR* (XCQ_05775). Kn^R^ | This study |
| pNPTS138-*acnD* | pNPTS138 derivative contacting the flanking regions of *prpR* (XCQ_05780). Kn^R^ | This study |
| pL-1 | pUFR034 derivative | This study |
| pL-2 | pBBR1MCS-2 derivative | This study |
| pL1-*PprpB*-mCherry-Flag | pL-1 derivative | This study |
| pL2-*prpR*-Flag | pL-2 derivative | This study |
| pMAL-c5x-*prpR* | pMAL-c5x derivative encoding *prpR* | This study |
