# Supplementary material for: Propionate serves as a degradable control agent of citrus canker by acidifying cytoplasm and depleting intracellular ATP in Xanthomonas citri
Source: mBio. 2025 Apr 29;16(6):e00642-25. doi: 10.1128/mbio.00642-25 (PMC12153269; doi:10.1128/mbio.00642-25)
Supplement: Table S2 — Primers. [file mbio.00642-25-s0005.docx]

Supplementary Table S2. Primer sequence used in this study.

| Primer name | Sequences |
| --- | --- |
| *For mutant construction* |  |
| *prpR*-LB-F | caattgaagccggctggcgccaagcttCGCGCCGAATCGCCGTATAAAC |
| *prpR*-LB-R  *prpR*-RB-F  *prpR*-RB-R  *prpB*-LB-F  *prpB*-LB-R  *prpB*-RB-F  *prpB*-RB-R  *prpC*-LB-F  *prpC*-LB-R  *prpC*-RB-F  *prpC*-RB-R  *acnD*-LB-F  *acnD*-LB-R  *acnD*-RB-F | tcgcgcttgccGAGTGTTTCAGCGTGAATCATTCGTTG  ctgaaacactcGGCAAGCGCGAACTTACACGAC  cgcgtcacggccgaagctagcgaattcCCACCATTGAAATCCAGGCATTCC  caattgaagccggctggcgccaagcttGTTCTTTGCCGGTGCCGGTTTC  gcaacgcgtcaGGCGGTTCCTTGGATGGGATGA  aaggaaccgccTGACGCGTTGCTGATCATCTTC  cgcgtcacggccgaagctagcgaattcCGCGCCTTGATATCGGCTTCTG  caattgaagccggctggcgccaagcttAGGTAATCGGCGCGATCAATGC  ctcatagcgacGCGATGAGCTCCCAAACTGTGT  gagctcatcgcGTCGCTATGAGGGCGATGCCGG  cgcgtcacggccgaagctagcgaattcCAATTCCACGCCGATGATGTCCG  caattgaagccggctggcgccaagcttCATCTTCATTGCCACCTCGCAC  aatcagcgctcGGCTGGCGATATCACAAGGCGG  tatcgccagccGAGCGCTGATTGAAACGCCCCA |
| *acnD*-RB-R | cgcgtcacggccgaagctagcgaattcCTCCAGTTGATCGATGACGTTGCC |
| *For qRT-PCR* |  |
| qgyrB-F  qgyrB-R  qhrpG-F  qhrpG-R  qhrpX-F  qhrpX-R  qhrpB1-F  qhrpB1-R  qprpB-F  qprpB-R  qalr-F  qalr-R  qkdpA-F  qkdpA-R  qpcaD-F  qpcaD-R  qtreS-F  qtreS-R  q18715-F  q18715-R  qvirB11-F  qvirB11-R  qrpsR-F  qrpsR-R  qrpoC-F  qrpoC-R  qcdyB-F  qcdyB-R | ATGACCGACGAACAAAACACCC  CGCCGACACGCCTTCTTCCTTG  GATCTTCGATGCCAGCTATGT  CGATACCAGGCCAGAATGTT  CGAACAAGCGTTACTGCTCTA  GAGATATCGTCGCTGACGTG  GGACTCACCCATGACAAGATTC  TACCGCGCTTGATGGAAATC  GCATCAACACGCTGGAAGAC  GGTGCGCTCGATGTTGAATG  GGATTCTTCGAACACGACGAG  CTTCAACCATACCCGCAACG  CTGATGGAAGGCAAGGAAGT  GAATCGTGCATCGCATTGAC  ATGGCTTATCTCCAGTTGCC  CAGCGAATTGCAGAAGGTCAG  GCCGAATGCCGAGATTGATA  CGATGAGTTGACGCTGGAAA  CACGATGAGCAATGATCCAGAC  GTGATGGTGATGGCGCTTTA  GTGCTGGGAATTCTGGACTATC  ATGGATCGTCTCCAGGTACA  TTGTCCGTGTACGGGATCAG  CTCACCGAGAACGGCAAGAT  TGATGGTTTCGGGCTTCTTC  GACCTGCTCAACCTCTTCAATC  CGTCTGACGTTGTGGATCAT  CCCTTGAGCACGCGATATAC |
| *For protein purification*  prpR-F  prpR-R  *For western blot*  PprpB::mCherry::Flag-F  PprpB::mCherry::Flag-R | atcgagggaaggatttcacatatgATTCACGCTGAAACACTCTTGAAAC  GCGAC AACGTCATGC  aattacctgcagggaattcggatccCTATGCACGCGCCGCCGCCTGC  tgtcgctgtacaagcatatgCGATACCGAATACACGCGGGTG  aggagctcgaattcggtaccCTACTTGTCATCGTCGTCCTTGTAG |
| *For EMSA*  EMSA-PprpB-F  EMSA-PprpB-R  EMSA-PprpC-F  EMSA-PprpC-R  EMSA-PacnD-F  EMSA-PacnD-R  EMSA-PprpF-F  EMSA-PprpF-R  EMSA-PprpB-F1-F  EMSA-PprpB-F1-R  EMSA-PprpB-F2-F  EMSA-PprpB-F2-R  EMSA-PprpB-F3-F  EMSA-PprpB-F3-R  EMSA-PprpB-F1 M1-F  EMSA-PprpB-F1 M1-R  EMSA-PprpB-F1 M2-F  EMSA-PprpB-F1 M2-R  EMSA-PprpB-F1 M3-F  EMSA-PprpB-F1 M3-R  EMSA-PprpB-F1 M4-F  EMSA-PprpB-F1 M4-R  EMSA-PprpB-F1 M5-F  EMSA-PprpB-F1 M5-R  *For 16S sequencing*  515f  806r | gagtgtttcagcgtgaatcattc  ggcggttccttggatgggatgac  gttgtttgcgcgcgaaggcgc  gcgatgagctcccaaactgtgtg  gtcgctatgagggcgatgccgg  ggctggcgatatcacaaggcgg  cccgcatggatgagcgaagcac  ggaacctcggttagctcactgg  gagtgtttcagcgtgaatcattcgttgcaattttgcaacaatcgatgcc  ggcatcgattgttgcaaaattgcaacgaatgattcacgctgaaacactc  ctgggtggtatgaaaatcaacgagttacgcttggcatggtgtttg  caaacaccatgccaagcgtaactcgttgattttcataccacccag  cggctgcacggtcatcccatccaaggaaccgcc  ggcggttccttggatgggatgaccgtgcagccg  gagtgtttcagcgtgaatcattcgattttgcaacaatcgatgcc  ggcatcgattgttgcaaaatcgaatgattcacgctgaaacactc  gagtgtttcagcgtgaatcattcgttgcaatttcaatcgatgcc  ggcatcgattgaaattgcaacgaatgattcacgctgaaacactc  gagtgtttcagcgtgaatcattcgccgcaattttgcaacaatcgatgcc  ggcatcgattgttgcaaaattgcggcgaatgattcacgctgaaacactc  gagtgtttcagcgtgaatcattcgttgcaattgcaacaatcgatgcc  ggcatcgattgttgcaattgcaacgaatgattcacgctgaaacactc  gagtgtttcagcgtgaatcattcgttgcaatttcctgcaacaatcgatgcc  ggcatcgattgttgcaggaaattgcaacgaatgattcacgctgaaacactc  GTGCCAGCMGCCGCGGTAA  GGACTACHVGGGTWTCTAAT |
